# Robust non-linear differential equation models of gene expression evolution across *Drosophila *development

**DOI:** 10.1186/1756-0500-5-46

**Published:** 2012-01-19

**Authors:** Alexandre Haye, Jaroslav Albert, Marianne Rooman

**Affiliations:** 1 BioSystems, BioModeling & BioProcesses Department, Université Libre de Bruxelles, CP 165/61, avenue Roosevelt 50, 1050 Bruxelles, Belgium

## Abstract

**Background:**

This paper lies in the context of modeling the evolution of gene expression away from stationary states, for example in systems subject to external perturbations or during the development of an organism. We base our analysis on experimental data and proceed in a top-down approach, where we start from data on a system's transcriptome, and deduce rules and models from it without *a priori *knowledge. We focus here on a publicly available DNA microarray time series, representing the transcriptome of *Drosophila *across evolution from the embryonic to the adult stage.

**Results:**

In the first step, genes were clustered on the basis of similarity of their expression profiles, measured by a translation-invariant and scale-invariant distance that proved appropriate for detecting transitions between development stages. Average profiles representing each cluster were computed and their time evolution was analyzed using coupled differential equations. A linear and several non-linear model structures involving a transcription and a degradation term were tested. The parameters were identified in three steps: determination of the strongest connections between genes, optimization of the parameters defining these connections, and elimination of the unnecessary parameters using various reduction schemes. Different solutions were compared on the basis of their abilities to reproduce the data, to keep realistic gene expression levels when extrapolated in time, to show the biologically expected robustness with respect to parameter variations, and to contain as few parameters as possible.

**Conclusions:**

We showed that the linear model did very well in reproducing the data with few parameters, but was not sufficiently robust and yielded unrealistic values upon extrapolation in time. In contrast, the non-linear models all reached the latter two objectives, but some were unable to reproduce the data. A family of non-linear models, constructed from the exponential of linear combinations of expression levels, reached all the objectives. It defined networks with a mean number of connections equal to two, when restricted to the embryonic time series, and equal to five for the full time series. These networks were compared with experimental data about gene-transcription factor and protein-protein interactions. The non-uniqueness of the solutions was discussed in the context of plasticity and cluster versus single-gene networks.

## Background

The impressive amount of data generated in the area of systems biology during the last few years, owing to powerful high-throughput technologies, has motivated novel bioinformatics and biomodeling developments to handle, rationalize and model these data. In the field of gene expression, DNA microarray techniques provide the simultaneous expression levels of many--sometimes all--genes in a cell sample, usually relative to those in a reference sample [1,2]. These data are extensively exploited to distinguish gene expression in pathological versus healthy cell systems, or in systems subject to different conditions or environments. Time series of DNA microarray data give a picture of the evolution of gene expression levels during, for example, the development stages of the host organism, the cell cycle, the circadian cycle, and the response to external perturbations; therefore they yield crucial dynamical information. In principle, the rationalization of these time-dependent data, if accurate and numerous enough, allows the reverse engineering of the gene network in the framework of a predefined mathematical model structure (see *e.g*. [3-12]). However, neither the uniqueness of the model structure nor the parameters that define it are guaranteed (see *e.g*. [13]).

A first possibility to handle the degeneracy of the solutions is to use *a priori *knowledge about the gene expression network, so as to limit the solution space. We take here a different approach, using biology-based constraints, and ask whether it could also reduce degeneracy. One biological constraint considered here is the robustness of the solutions with respect to parameter variations (see *e.g*. [14-16]). Indeed, all biological systems have a stochastic behavior, where the changes in the environment, the varying amount of biomolecules present, their non-deterministic binding and function, etc., do not affect the main properties of the system, which continues to give similar response to the same stimuli. Only very large or very specific perturbations can lead the system out of its correctly functioning state, and lead it to another state or cause dysfunction and illnesses. It is thus of extreme importance that the models that simulate biological systems have the same properties, and thus do not yield very different solutions for similar parameter values.

Another biological constraint is related to the stability of the solutions when extrapolated in time. Even though the available data usually cover only a part of the system's life, it is reasonable to assume that the expression levels continue to be of the same order of magnitude, never becoming unrealistically large or negative. The same property is expected to be built in the model: the solutions must take realistic values until the next perturbation, development stage, or the end of the organism's life.

We analyze in this paper the effects of adding these biological constraints on the modeled dynamics of gene expression, particularly in the framework of the development of an organism. More specifically, we investigate whether these constraints limit the choice of the model structure and/or its parameter values. We use *Drosophila *as model organism, and model the time evolution of its transcriptome using coupled, linear and non-linear, differential equations.

## Methods

### DNA microarray time series

With the DNA microarray technique [1,2] one measures the fluorescence intensities *I_μ _*(*τ*) emitted by the fluorophores attached to the mRNAs labeled here by *μ *(more precisely, to the corresponding cRNAs or cDNAs), which are extracted from a specific sample taken at a given time *τ *and are hybridized to their complementary sequence attached to a microarray. These intensities are usually expressed relative to the intensity $I_{\mu }^{R}$ of the same mRNAs taken from a reference sample. As the measures come from different hybridizations, they must be normalized to correct for different effects including the unequal quantities of RNA copies, differences in labeling or detection efficiencies between the fluorescent dyes, and systematic biases in the measured expression levels [17,18]. We define each gene expression profile *X_μ _*(*τ*) as a function of the normalized intensities , that is:

(1) $$X_{\mu }\left(\tau \right)=\frac{{Ĩ}_{\mu }\left(\tau \right)}{{Ĩ}_{\mu }^{R}}.$$

Time series are obtained when considering the sample at *N *different time points *τ_i _*(*i *= *1*,..*N*). We made here the common assumption that the RNA concentrations and fluorescence intensities are proportional [19], *i.e*. that *X_μ _*(*τ*) represents the RNA concentration up to a gene-dependent scaling factor $I_{\mu }^{R}$. In what follows, the index *μ *will refer indistinguishably to the gene product--RNA or protein--or the gene wherein the gene product is encoded.

We use here a DNA microarray time series of male *Drosophila melanogaster *[20]. It contains the expression levels of 4,028 genes across all four developmental phases. Of the 67 time points, 31 are in the embryonic phase (covering 24 h), 10 in the larval phase (81 h), 18 in the pupal phase (111 h), and 8 in the adult phase (30 days). The reference sample consists of a mixture of all samples of the series, *i.e*. of *Drosophila *of all ages. We considered here on the one hand the complete time series of 67 time points, and on the other hand the part of the time series covering the embryonic phase, which contains the 31 first time points.

### Classification of gene expression profiles

It is technically impossible to model the evolution of the expression levels of thousands of genes, given the few data points available. Moreover, even if we had a sufficient number of time points to ensure parameter identification, the problem would be degenerate, in that multiple solutions with almost the same ability to reproduce the data would exist. Indeed, many of the gene expression profiles are very similar and are thus basically indistinguishable without additional information. We therefore cluster the gene expression profiles into a limited number of distinct classes.

The clustering is performed on the basis of the least-square distance *D *[21]. This distance is translation-invariant and scale-invariant with scaling dimension 1/2. This means that the distance *D *between two profiles *X_μ _*(*τ*) and *X_v _*(*τ*) satisfies, $\forall a,b\in ℜ:D\left(X_{\mu },X_{v}+b\right)=D\left(X_{\mu },X_{v}\right)$ and $D\left(X_{\mu },a.X_{v}\right)=\sqrt{a}D\left(X_{\mu },X_{v}\right)$. The choice of this distance is justified by the fact that expression levels are generally defined relative to a gene-dependent but time-independent reference expression level (see Eq. 1). The scaling factor between two profiles may thus simply be due to their different reference expression levels, and thus has no intrinsic meaning. Moreover, we chose not to take into account the difference between two profiles with the same shape but different average expression levels, as such profiles are merely translated with respect to each other. This distance has proven to be relevant for detecting the limits of developmental stages or perturbations phases from DNA microarray data [21].

With these constraints of scale and translation invariance and the usual symmetry constraint $D\left(X_{\mu },X_{v}\right)=D\left(X_{v},X_{\mu }\right)$, the distance *D *is shown to be of the form [21]:

(2) $$D\left(X_{\mu },X_{v}\right)=\sqrt{\frac{\varsigma_{\mu }\varsigma_{v}}{N}\overset{N}{\underset{k=1}{\sum }}{\left(\frac{X_{\mu }\left(\tau_{k}\right)-\langle X_{\mu }\rangle }{\varsigma_{\mu }}\pm \frac{X_{v}\left(\tau_{k}\right)-\langle X_{v}\rangle }{\varsigma_{v}}\right)}^{2}},$$

in terms of the mean $\langle X_{\mu }\rangle$ and standard deviation *ς_μ_*:

(3) $$\langle X_{\mu }\rangle =\frac{1}{N}\overset{N}{\underset{k=1}{\sum }}X_{\mu }\left(\tau_{k}\right)\;\text{and}\;\varsigma_{\mu }=\sqrt{\frac{1}{N}\overset{N}{\underset{k=1}{\sum }}{\left(X_{\mu }\left(\tau_{k}\right)-\langle X_{\mu }\rangle \right)}^{2}},$$

where the sign that minimizes *D *is chosen in Eq. 2.

Based on this distance, the gene expression profiles are clustered using a hierarchical, tree-like algorithm. It starts by considering each gene as a class on its own. It then joins, at each step, the two classes for which the average distance *D *between any pairs of profiles from the two classes is minimum. It stops when all genes are in the same class. This clustering tree is then cut at a certain level by putting a threshold on the maximum number of classes, denoted *C*. The choice of this threshold is always a subjective matter and depends on the aim of the clustering. Here, the number of classes must be sufficiently low to ensure that they are manageable for modeling purposes. Moreover, to have a meaningful classification, the distances between profiles within each cluster must be sufficiently low and those between profiles of different clusters sufficiently high.

Each of the *C *clusters labeled by *c *(*c *= 1,...,*C*) is represented the average profile ${\bar{X}}_{c}\left(\tau \right)$. To compute this profile, we first identified the representative profile of the cluster, defined as the profile for which the distance with respect to all other members of the class is minimum. All the profiles of the cluster were then superimposed on the representative using the translation and scaling factor that minimize the distance. The average profile ${\bar{X}}_{c}\left(\tau \right)$ corresponds then to the average, at each time point, of all translated and scaled profiles in the cluster.

### Model structures

The system of differential equations that correctly models the evolution of gene expression across development stages is not known, and even less is known about equations that can model gene clusters. We therefore test several model structures. Assuming the system to be autonomous, we consider structures of the form:

(4) $${\dot{\bar{X}}}_{c}\left(t\right)=\Theta_{c}\left(\bar{\text{X}}\right)-\Delta_{c}\left(\bar{X}\right){\bar{X}}_{c}\left(t\right),$$

where $\bar{X}=\left({\bar{X}}_{1},\ldots {\bar{X}}_{C}\right)$ and *t *is the real, continuous time. The dot means the derivative with respect to *t*. Since the transcription term $\Theta_{c}\left(\bar{X}\right)$ is defined to be positive, it increases the concentration ${\bar{X}}_{c}$ of cluster *c*, basically through the binding of transcription factors, which either activate or repress genes in this cluster. The positively defined function $\Delta_{c}\left(\bar{X}\right)$ is called the degradation factor because it describes the degradation, destabilization or inhibition of the activity of the gene products belonging to cluster *c*, or their removal from the system. Note that this general model, which is deterministic, represents the average behavior of the system, which is stochastic.

Five model structures are studied. The first is the linear model, defined as:

(5) $$m_{lin}:\;\Theta_{c}\left(\bar{X}\right)=\overset{C}{\underset{d=1}{\sum }}M_{cd}{\bar{X}}_{d}\left(t\right),\;\Delta_{c}\left(\bar{X}\right)=0.$$

The other four model structures are non-linear. They resemble models that have been developed to describe *Escherichia coli *subject to glucose-lactose diauxie [11]. The first reads as:

(6) $$m_{NC}^{pol}:\;\Theta_{c}\left(\bar{X}\right)=\rho_{c}\frac{\overset{C}{\underset{d=1}{\sum }}A_{cd}{\bar{X}}_{d}\left(t\right)}{\left(1+\overset{C}{\underset{d=1}{\sum }}A_{cd}{\bar{X}}_{d}\left(t\right)\right)\left(1+\overset{C}{\underset{d=1}{\sum }}B_{cd}{\bar{X}}_{d}\left(t\right)\right)},\;\Delta_{c}\left(\bar{X}\right)=\gamma_{c},$$

where *A_cd_*, *B_cd_*, *ρ_c_*, *γ_c_*≥ 0. The degradation factor is considered to be constant. The parameters *A_cd _*weigh the effect of activators on the expression of genes from cluster c, whereas *B_cd _*weigh the effect of repressors. The transcription term is thus proportional to the product of the probability that an activator is bound to the promoter and the probability it is not bound to a repressor. It is obtained by making the approximation that the expression of a gene can be activated or repressed by a single protein, and does not require protein complexes or cascades of interacting proteins. Another assumption is that the form of the dynamic equations remains the same for individual genes and for gene clusters.

The degradation term can also be considered as dependent on gene expression levels. As in the model describing diauxie [11], we chose it to be of the form:

(7) $$m_{CN}^{\exp}:\;\Theta \left(\bar{X}\right)=\rho_{c},\;\Delta \left(\bar{X}\right)=\frac{\kappa_{c}^{+}+\kappa_{c}^{-}\exp\left(\overset{C}{\underset{d=1}{\sum }}K_{cd}{\bar{X}}_{d}\left(t\right)\right)}{1+\exp\left(\overset{C}{\underset{d=1}{\sum }}K_{cd}{\bar{X}}_{d}\left(t\right)\right)}.$$

with $\rho_{c},\;\kappa_{c}^{+},\;\kappa_{c}^{-}\geq 0$. It was assumed here that the degradation factor is modulated by interactions between gene products so as to either prolong (*e.g*. through stabilizing complexes) or shorten (*e.g*. through degradation by proteases) their period of activity. The two parameters $\kappa_{c}^{+}$ and $\kappa_{c}^{-}$ symbolize the maximum and minimum degradation rate when $\kappa_{c}^{+}>\kappa_{c}^{-}$ and the converse when $\kappa_{c}^{+}<\kappa_{c}^{-}$, and *K_cd _*gives the influence (stabilizing or destabilizing according to its sign) of gene product *d *on gene product *c*.

The above expression for the degradation term may also be used for the transcription term, while keeping the degradation factor constant. This yields:

(8) $$m_{NC}^{\exp}:\;\Theta \left(\bar{X}\right)=\frac{\lambda_{c}^{+}+\lambda_{c}^{-}\exp\left(-\overset{C}{\underset{d=1}{\sum }}L_{cd}{\bar{X}}_{d}\left(t\right)\right)}{1+\exp\left(-\overset{C}{\underset{d=1}{\sum }}L_{cd}{\bar{X}}_{d}\left(t\right)\right)},\;\Delta \left(\bar{X}\right)=\gamma_{c}.$$

with $\gamma_{c},\;\lambda_{c}^{+},\;\lambda_{c}^{-}\geq 0$.

Finally, the last model structure we considered has the same expression for both the transcription term and the degradation factor:

(9) $$m_{NN}^{\exp}:\;\Theta \left(\bar{X}\right)=\frac{\lambda_{c}^{+}+\lambda_{c}^{-}\exp\left(-\overset{C}{\underset{d=1}{\sum }}L_{cd}{\bar{X}}_{d}\left(t\right)\right)}{1+\exp\left(-\overset{C}{\underset{d=1}{\sum }}L_{cd}{\bar{X}}_{d}\left(t\right)\right)},\;\Delta \left(\bar{X}\right)=\frac{\kappa_{c}^{+}+\kappa_{c}^{-}\exp\left(-\overset{C}{\underset{d=1}{\sum }}K_{cd}{\bar{X}}_{d}\left(t\right)\right)}{1+\exp\left(-\overset{C}{\underset{d=1}{\sum }}K_{cd}{\bar{X}}_{d}\left(t\right)\right)}.$$

with $\kappa_{c}^{+},\;\kappa_{c}^{-},\;\lambda_{c}^{+},\;\lambda_{c}^{+}\geq 0$.

### Network identification

To manage the large amount of parameters and the non-linearity of the equations, we used a two-stage procedure for parameter identification. The first stage consists of reproducing the derivatives of the gene expression levels rather than the gene expression levels themselves. This entails considering the expression levels and their derivatives as independent variables and reducing the identification to an algebraic problem, where the functions *ζ_c _*to be minimized are decoupled. These read as:

(10) $$\zeta_{c}\left(J_{c}\right)=\sqrt{\frac{1}{N}\overset{N}{\underset{k=1}{\sum }}{\left({\dot{\bar{X}}}_{c}\left(\tau_{k}\right)-{\hat{\dot{\bar{X}}}}_{c}\left(\tau_{k},J_{c}\right)\right)}^{2}}\;\text{and}\;\zeta_{c}\left(J_{c}\right)=\sqrt{\frac{1}{C}\overset{C}{\underset{c=1}{\sum }}\zeta_{c}{\left(J_{c}\right)}^{2}}.$$

The estimates of the *t*-derivative of the gene expression profiles, referred to as ${\hat{\dot{\bar{X}}}}_{c}$, are obtained from the right hand side of Eq. 4, with the transcription term and degradation factor given by one of the model structures defined by eqs (5-9). The parameters entering these equations are generically denoted as **J**_c_. The *t*-derivatives of the measured gene expression profiles, ${\dot{\bar{X}}}_{c}$, are obtained by simple data interpolation with the cubic smoothing splines algorithm *csaps *in Matlab (The MathWorks, Inc., Natick, MA, USA).

The procedure used to identify the parameters **J_c _**is inspired by [22], and works as follows. The connectivity *q *is defined to be the average number of connections that end at a node of the network. The number of parameters defining a connection depends on the model structure. In a first step *q *is considered identical for all nodes, that is, an identical number of gene classes regulates each gene class. We start by putting *q *= 1 and test, for each gene cluster *c*, all possible connections one by one. The identification of the parameters that define each connection and minimize *ζ_c _*is first performed using the global optimization algorithm *Direct *[23] implemented in Matlab. The parameters are restricted, in absolute value, to the [10] interval, corresponding roughly to the range of values adopted by ${\bar{X}}_{c}$, to ensure their biological significance. The solution obtained by this algorithm is then refined: it is used to initialize the local optimization algorithm *fmincon *of Matlab. For each cluster *c*, the connection for which *ζ_c _*is minimum is kept. This procedure is repeated for *q *= 2 up to *q *= C. Note that each time a connection is added, the parameters defining the previously fixed connections are freed and reoptimized.

### Parameter identification

In the second stage, the parameters that maintain the network defined in the previous stage and minimize the difference between measured and estimated profiles, rather than their derivatives, are identified. More precisely, we start with the connections determined in the *q *= 1 solution of the previous stage, with the parameters initialized either to the values of this solution or to zero, whichever minimizes the standard deviation σ, defined as:

(11) $$\sigma_{c}\left(J\right)=\sqrt{\frac{1}{N}\overset{N}{\underset{k=1}{\sum }}{\left({\bar{X}}_{c}\left(\tau_{k}\right)-{\hat{\bar{X}}}_{c}\left(\tau_{k},J\right)\right)}^{2}}\;\text{and}\;\sigma_{c}\left(J\right)=\sqrt{\frac{1}{C}\overset{N}{\underset{c=1}{\sum }}\sigma_{c}{\left(J_{c}\right)}^{2}},$$

where **J **= (**J**_1_,..., **J**_C_). We then free the parameters and optimize them using the *fmincon *optimization algorithm of Matlab, so as to minimize the function σ. The estimate of the gene expression profiles, ${\hat{\bar{X}}}_{c}$, is obtained by integration of the differential equations (4), using one of the model structures given by eqs (5-9). Note that the equations for different clusters are no longer decoupled as they were in the first stage. Thus both σ*_c _*and ${\hat{\bar{X}}}_{c}$ depend on **J **rather than on **J**_c _only. We then repeat this procedure by choosing the *q *= 2 up to *q *= *C *solutions obtained in the first stage, freeing the parameters and identifying them by minimizing the function (11). The initial values of the parameters are chosen to be those obtained for the *q*-1 identification, with the newly added parameters set to zero.

In practice, we do not continue this procedure up to *q *= *C*, but stop it when the value of σ stops decreasing significantly, thus when no additional connection improves significantly the quality of the data reproduction.

### Parameter reduction

The next step consists of eliminating unnecessary parameters among *M_cd_*, *A_cd_*, *B_cd_*, *L_cd_*, and *K_cd _*that appear in eqs (5-9). We require that at least one connection per gene class be kept. We proceed by dropping one parameter at a time, according to different criteria detailed in what follows. Note that we also tried to drop several parameters at the same time, but the results were worse. The reduction procedure was stopped when the value of σ exceeded 0.5, as the measured and estimated gene expression profiles started to differ too much.

Several reduction procedures were tested. They consist of eliminating at each iteration:

1) the parameter of smallest absolute value (this procedure will be referred to as Ψ*_v_*);

2) the parameter which, when dropped, leads to the smallest increase of σ (Ψ*_σ_*);

3) the parameter that is most sensitive to a perturbation (Ψ*_P_*) (in order to determine this parameter, we add or subtract to each parameter in turn 1% of its value, estimate again the gene expression profile and compute the resulting σ-value; the eliminated parameter is the one that leads to the largest increase in σ upon perturbation);

4) the least sensitive parameter in the Fisher sense $\Psi_{F}^{-}$;

5) the most sensitive parameter in the Fisher sense $\Psi_{F}^{+}$.

To determine the most or the least sensitive parameter in the Fisher sense, we compute the Fisher information matrix **F **[24], defined from the change of the estimated profiles upon infinitesimal variations of the parameters:

(12) $$F_{ij}=\overset{C}{\underset{c=1}{\sum }}\overset{N}{\underset{k=1}{\sum }}\left(\frac{\partial {\hat{\bar{X}}}_{c}\left(J_{c},\tau_{k}\right)}{\partial J_{i}}\frac{\partial {\hat{\bar{X}}}_{c}\left(J_{c},\tau_{k}\right)}{\partial J_{j}}\right),$$

where *i*, *j *= 1,..*p*, with *p *being the total number of parameters. The parameter *i *to be eliminated is the one that is correlated with at least one other parameter *j*, *i.e*. $\exists j:F_{ij}\geq 0.9\sqrt{F_{ii}F_{jj}}$, and is the least sensitive (in point 4) or the most sensitive (in point 5), *i.e*. corresponds to the minimum value of *F_ii _*(in point 4) or its maximum value (in point 5).

After a parameter is eliminated the remaining parameters are optimized again using the local optimization algorithm *fmincon*. The elimination procedure is then reiterated. Note that the reductions 1, 2 and 4 are standard procedures, whereas the reductions 3 and 5 attempt to eliminate parameters that are sensitive to perturbations.

### Evaluation of the solutions

Four criteria were used to evaluate the quality of the estimated profiles:

1) the number or remaining parameters;

2) the standard deviation σ between estimated and experimental profiles, defined in Eq. 11;

3) the robustness of the solution with respect to perturbations of its parameters; this is estimated by adding to each parameter in turn ± 1% of its value, determining which perturbation leads to the largest deviation between measured and estimated expression levels, $\left|{\bar{X}}_{c}\left(\tau_{k}\right)-{\hat{\bar{X}}}_{c}\left(\tau_{k}\right)\right|$, for any cluster *c *and time point *τ_k_*, and computing the value of the standard deviation *σ *obtained with this perturbed parameter, denoted *σ_pert_*;

4) the stability of the solution, evaluated by extrapolating the estimated profiles up to a time *τ_end _*and by computing the difference between the average value of the estimated gene expression levels over the measuring period and the extrapolated level:

(13) $$\chi =\overset{C}{\underset{c=1}{\sum }}\left|\left(\frac{1}{N}\overset{N}{\underset{k=1}{\sum }}{\hat{\bar{X}}}_{c}\left(\tau_{k}\right)\right)-{\hat{\bar{X}}}_{c}\left(\tau_{end}\right)\right|.$$

The time *τ_end _*corresponds to 3 times the measured time span and at most the *Drosophila *life time, *i.e*. 80 days.

## Results and discussion

### Gene clusters

The 4,028 gene expression profiles across *Drosophila *development, presented in section 2.a, are classified using a translation-invariant and scale-invariant distance measure and a hierarchical tree-like classification procedure, as detailed in section 2.b. To obtain the final classes, we cut the clustering tree by putting a threshold on the maximum number of classes, so as to ensure that the distances between profiles within each cluster are sufficiently low, that the distances between profiles of different clusters are sufficiently high, and that the number of classes is sufficiently low for allowing the identification of the models' parameters on the basis of the available data points. Taking these criteria into account, we took the number of classes *C *to be equal to 12 for the full time series and 10 for the embryonic stage.

The clusters for the embryonic stage and for the complete time series are shown in Additional file 1: Figures S1a-b; the members of each cluster are given in Additional file 1: Table S1 a-b. Each cluster is represented by the average profile, ${\bar{X}}_{c}\left(\tau_{k}\right)$, defined at each time point as the average of the gene expression levels of the members of the class, after suitable scaling and translation on the representative profile (see section 2b). The average profiles are much smoother than the individual profiles, and can be considered as relatively noise-free. In what follows we will focus on the average profiles and model their evolution using the five structures described by eqs (5-9).

Note that our clustering procedure is based on a distance measure between profiles that is adapted to our modeling purposes, and that it differs from previously described ones [20,25]. In particular, we clustered the gene expression levels *X_μ _*(*τ*) defined in Eq. 1 rather than their logarithms, because these levels are the naturally appearing functions in our differential equation model given in Eq. 4.

### Cluster network identification

**Figure 1:**
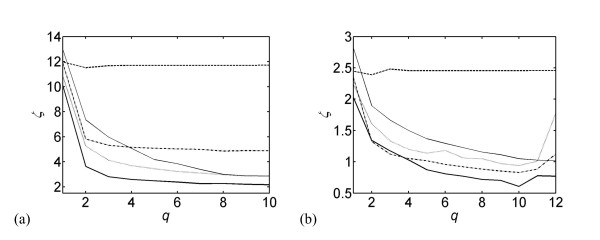
**Bottom-up construction of the gene expression network**. The standard deviation *ζ *is given as a function of the connectivity *q*. The five curves correspond to the five model structures of eqs (5-9): dashed line: $m_{NC}^{pol}$; thick solid line: $m_{NN}^{\exp}$; dashed-dotted line: $m_{NC}^{\exp}$; dotted line: $m_{CN}^{\exp}$; thin solid line: *m_lin_*. (a) embryonic time series; (b) full time series.

The first stage of parameter identification is a bottom-up procedure devised to fix the gene expression network. It starts with a single connection per cluster and ends with a constant number of connections *q *per cluster, as described in section 2.d. This algebraic procedure attempts to minimize *ζ*, *i.e*. the standard deviation between the time derivatives of estimated and experimental gene expression profiles (Eq. 10). The procedure is stopped when *ζ *does not significantly decrease any more. The results are given in Figures 1a-b for the embryonic and full time series.

The value of *ζ*, for the same connectivity *q*, is higher for the embryonic stage than for the full time series. This is due to the fact that in the embryonic stage the profiles are less smooth and thus the derivatives are higher than in the full time series. The model structure $m_{NC}^{pol}$, expressed in terms of polynomials (Eq. 6), fails to reproduce the gene expression profiles for both the embryonic stage and full time series, with *ζ*-values remaining almost constant as the connectivity increases. The best structure turns out to be $m_{NN}^{\exp}$, with the transcription term and the degradation factor having exponential forms (Eq. 9). Note that these two model structures have almost the same number of parameters; therefore the drop in performance cannot be due to this difference. However, all the parameters of $m_{NC}^{pol}$ are required to be positive, which could be problematic in the parameter identification. The other three model structures, which have significantly less parameters, minimize *ζ *reasonably well.

### Parameter identification

**Figure 2:**
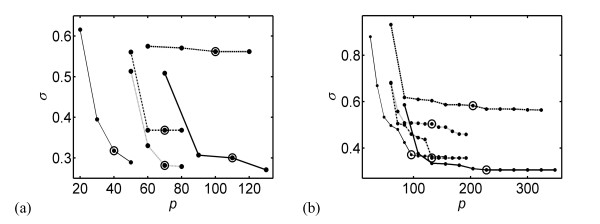
**Parameter optimization for the gene expression networks**. The standard deviation *σ *is given as a function of the total number of parameters *p*. The five curves correspond to the five model structures of eqs (5-9): dashed line: $m_{NC}^{pol}$; thick solid: $m_{NN}^{\exp}$; dashed-dotted line: $m_{NC}^{\exp}$; dotted line: $m_{CN}^{\exp}$; thin solid line: *m_lin_*. The number of parameters (*p*) depends on the connectivity per class (*q*) and the number of classes (*C*): *p *= (2*q*+4)*C *for $m_{NC}^{pol}$, *p *= (2*q*+5)*C *for $m_{NN}^{\exp}$, *p *= (*q*+4)*C *for $m_{NC}^{\exp}$ and $m_{CN}^{\exp}$, and *p *= (*q*+1)*C *for *m_lin_*. The value of the minimum connectivity *q^m ^*necessary to yield good data reproduction is indicated by a large circle. (a) Embryonic time series; the number of parameters *p *shown corresponds to connectivities *q *between 1 and 4; *q^m ^*= 3; (b) full time series; the number of parameters *p *shown corresponds to connectivities *q *between 1 and 12; *q^m ^*= 7.

Having fixed the gene expression network, the second step consists of identifying the parameters that minimize *σ*, *i.e*. the standard deviation between estimated and measured gene expression profiles (Eq. 11), rather than their time derivatives. The results are given in Figures 2a-b for the embryonic and full time series.

The results confirm those obtained in the previous step: the model structure $m_{NC}^{pol}$ does worse than all the others. The other four structures do reasonably well. The structure with the largest number of parameters, $m_{NN}^{\exp}$, does well in both stages, and so does the linear model, *m_lin_*, which has by far the fewest parameters. Interestingly, the two structures $m_{CN}^{\exp}$ and $m_{NC}^{\exp}$, which have the same number of parameters but have, respectively, the transcription term and the degradation factor constant, behave differently in the embryonic and full time series: the former does better in the embryonic stage and the latter in the full series.

Based on these results, we determined the minimum connectivity *q^m ^*that must be considered to yield a fair reproduction of the expression profiles, and beyond which the reproduction does not significantly improve. By visual inspection of Figures 2a-b, we determined that *q^m ^*= 3 for the embryonic stage and *q^m ^*= 7 for the full time series. Clearly, the network needs more connections to describe reliably the complete time series than just the embryonic stage.

### Evaluation of the solutions

**Figure 3:**
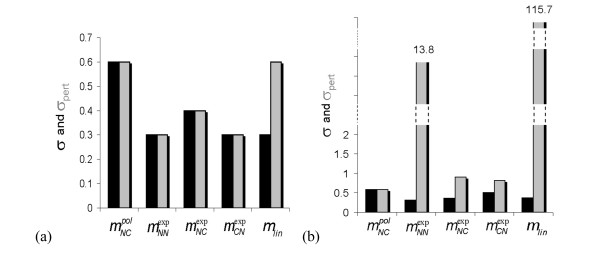
**Gene profile reproduction and robustness of the solutions**. Black bars indicate the standard deviation *σ *and grey bars the standard deviation *σ*_pert _after the parameter perturbations defined in section 2g. The five bars correspond to the five model structures of eqs (5-9). (a) Embryonic time series; (b) full time series.

The solutions obtained with these values of *q^m ^*are evaluated according to the criteria listed in section 2.g. As seen in Tables 1, 2 and Figures 3a-b, all the model structures except for $m_{NC}^{pol}$ allow a good reproduction of the data, with *σ*-values below 0.5. The linear model *m_lin _*achieves this with the smallest number of parameters. This result would *a priori *push to the selection of the linear model *m_lin _*and to the rejection of the non-linear model $m_{NC}^{pol}$.

**Table 1:** Characteristics of full and reduced estimated solutions for the embryonic time series.

*Model*	*Reduction*	σ	σ*_pert_*	χ	*p*	*q*
*m_lin_*	-	**0.32**	**0.56**	**200.3**	**40**	**3**

$m_{NC}^{pol}$	-	0.56	0.56	5.3	100	3

$m_{NC}^{\exp}$	-	**0.37**	**0.37**	**4.8**	**70**	**3**

$m_{NN}^{\exp}$	-	**0.30**	**0.30**	**4.1**	**110**	**3**
	
	Ψ***_σ_***	**0.31**	**0.34**	**3.6**	**78**	**2.1**
	
	Ψ***_v_***	**0.32**	**0.35**	**3.9**	**85**	**2.3**
	
	**$\Psi_{F}^{-}$**	**0.31**	**0.33**	**2.7**	**101**	**3**
	
	$\Psi_{F}^{+}$	**0.30**	**0.32**	**4.9**	**108**	**3**
	
	Ψ*_P_*	**0.33**	**0.38**	**7.2**	**108**	**3**

$m_{CN}^{\exp}$	-	**0.28**	**0.31**	**2.1**	**70**	**3**
	
	Ψ*_σ_*	**0.28**	**0.42**	**1.5**	**65**	**2.5**
	
	Ψ*_v_*	**0.29**	**0.37**	**1.3**	**62**	**2.2**
	
	$\Psi_{F}^{-}$	**0.28**	**0.33**	**2.0**	**69**	**2.9**
	
	$\Psi_{F}^{+}$	**0.28**	**0.31**	**1.5**	**69**	**2.9**
	
	Ψ*_P_*	0.35	0.75	4.0	69	2.9

The solutions in bold satisfy the condition *σ_c_*≤0.5 ∀*c*. The gray lines correspond to the selected solutions whose network is depicted in Figure 6.

**Table 2:** Characteristics of full and reduced estimated solutions for the complete time series.

*Model*	*Reduction*	σ	σ*_pert_*	χ	*p*	*q*
*m_lin_*	-	0.37	115.70	41.9	96	7

$m_{NC}^{pol}$	-	0.58	0.58	1	216	7

$m_{CN}^{\exp}$	-	**0.50**	**0.81**	**1.47**	**132**	**7**

$m_{NN}^{\exp}$	-	**0.31**	**13.77**	**0.7**	**228**	**7**
	
	Ψ***_σ_***	**0.36**	**9.08**	**0.1**	**161**	**6.1**
	
	Ψ***_v_***	**0.27**	**9.12**	**2.4**	**188**	**6.7**
	
	**$\Psi_{F}^{-}$**	**0.32**	**1.68**	**0.8**	**187**	**6.8**
	
	$\Psi_{F}^{+}$	1.43	0.91	1.0	131	7
	
	Ψ*_P_*	0.88	0.88	0.1	225	7

$m_{NC}^{\exp}$	-	**0.36**	**0.90**	**1.0**	**132**	**7**
	
	Ψ*_σ_*	**0.34**	**1.08**	**0.9**	**124**	**6.3**
	
	Ψ*_v_*	**0.34**	**1.02**	**0.8**	**108**	**5**
	
	$\Psi_{F}^{-}$	**0.35**	**0.99**	**1.0**	**127**	**6.6**
	
	$\Psi_{F}^{+}$	**0.34**	**0.81**	**1.0**	**122**	**6.2**
	
	Ψ*_P_*	0.65	0.65	0.1	131	6.9

The solutions in bold satisfy the condition *σ_c_*≤0.5 ∀*c*. The gray lines correspond to the selected solutions whose network is depicted in Additional file 1: Figure S5.

However, when perturbing the parameters by ± 1%, the linear model appears to be by far the least robust. This is particularly visible for the full time series where the perturbed *σ *values are 300 times larger than the unperturbed ones. Note that the non-linear $m_{NC}^{\exp}$ model structure also lacks robustness for certain parameter variations in the case of the full series.

**Figure 4:**
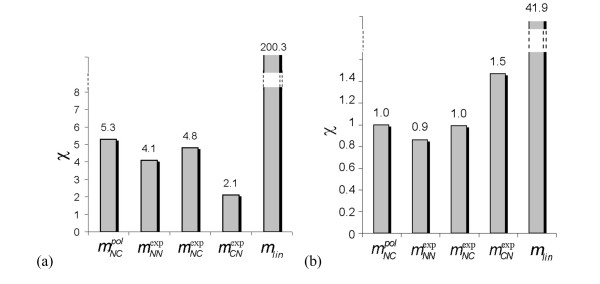
**Stability of the solutions upon extrapolation in time**. Bars indicate the value of χ, which corresponds to the difference between the estimated expression levels averaged over the time points and the expression level extrapolated to *τ_end _*(see Eq. 13). The five bars correspond to the five model structures of eqs (5-9). (a) Embryonic time series; *τ_end _*corresponds to 3 times the measuring period; (b) full time series; *τ_end _*corresponds to the *Drosophila *life time, *i.e*. 80 days.

Furthermore, the stability of the solutions, which is evaluated by extrapolating the estimated profiles in time (see Eq. 13), is depicted in Figures 4a-b. We consider that a solution is stable if the expression levels at extrapolated times are of the same order of magnitude as the average level. We observe that only the linear model displays a very poor stability. All non-linear models appear reasonably stable.

The principal criterion that the solutions have to fulfill is the reproduction of the data. However, to ensure biological significance, they must moreover be reasonably robust against parameter perturbations (see *e.g*. [14-16]). Indeed, the gene expression process in real cells is intrinsically stochastic, but gives nevertheless basically the same response whatever the system's perturbations are. If the system were not robust, any perturbation would lead to dysfunctional cells. Moreover, the modeled profiles must also be relatively stable in the extrapolated time regime, until the end of a development stage or the organism's life, in order to keep the levels of expression in a realistic range.

Note that network models involving individual genes for which a few specific parameters are sensitive to perturbations may not be immediately disqualified. However, we do not work here with networks of individual genes, but rather with clusters containing hundreds of genes. Therefore, if a parameter that represents the strength of the interaction between these groups of genes is sensitive to perturbations, it is not one but a large number of genes that deviate from their intended expression profiles. We thus require our models to be robust with respect to all (tested) parameter variations.

Hence we can conclude that the non-linear model $m_{NC}^{pol}$ is inappropriate as it fails in reproducing the expression profiles, and that the linear model *m_lin _*is unsuitable as it is non-robust and non-stable. Only the three non-linear structures $m_{NN}^{\exp}$, $m_{NC}^{\exp}$ and $m_{CN}^{\exp}$ will be further analyzed.

### Parameter reduction

In the identification stage described in sections 3.b-c, we determined the number of connections *q^m ^*per gene necessary to minimize *σ*. However, some of the genes probably require fewer connections than others, and some of the connections fewer parameters. To identify the parameters that may be dropped without altering the data reproduction too much, we applied the five different reduction schemes described in section 2f. Three of them are commonly used schemes: Ψ*_v _*drops the parameters of smallest absolute value; Ψ*_σ_*, the parameters that increase *σ *the least; and $\Psi_{F}^{-}$, the parameters that are correlated with at least one other parameter and are the least sensitive to infinitesimal parameter variations as defined by the Fisher matrix. The latter two reductions, $\Psi_{F}^{-}$ and Ψ*_P_*, aim at selecting solutions that are the most robust with respect to variations of the parameters: they drop parameters that are the most sensitive to infinitesimal and finite parameter variations, respectively. Note that the parameters are dropped one by one in all these schemes. The scores reached, when dropping several parameters simultaneously, are not as good.

The quality of the data reproduction (*σ*), the robustness (*σ_pert_*) and the stability upon extrapolation (χ) of the reduced solutions obtained by the five different reduction procedures applied to the three remaining model structures $m_{NN}^{\exp}$, $m_{NC}^{\exp}$ and $m_{CN}^{\exp}$, for the embryonic and full time series, are given in Additional file 1: Figure S2. As expected, the procedure Ψ*_σ_*, which drops the parameters that increase *σ *the least, leads almost invariably to the solutions that best reproduce the data. The procedure Ψ*_v_*, which drops the parameters of smallest absolute value, does also very well in this respect, whereas the other three procedures generally perform less well. Surprisingly, the reduced solutions obtained by the procedures Ψ*_σ _*and Ψ*_v _*are also robust against perturbations and stable upon extrapolation, usually even more than the solutions obtained with the procedures $\Psi_{F}^{+}$ and Ψ*_P _*that are nevertheless designed to select robust solutions. The commonly used Fisher matrix-based $\Psi_{F}^{-}$ procedure that keeps sensitive parameters is in general not as good as the Ψ*_σ _*and Ψ*_v_*, for none of the criteria considered, and is of the same order as $\Psi_{F}^{+}$ and Ψ*_P_*. Note that, in some cases, $\Psi_{F}^{+}$ and $\Psi_{F}^{-}$ give very similar results although they seem *a priori *quite different. However, it has to be reminded that they have a common part: they both drop correlated parameters, which may explain the similarity.

We now proceed to select the best reduced solutions. Up to now we used the criterion for data reproduction to be *σ*≤0.5. However, the value of *σ *is an average over all clusters, so that this value can be reached when all profiles are well reproduced, but also when almost all profiles are well reproduced and a few are not. To prevent this from happening, we define a new criterion, *σ*_χ_≤0.5 ∀c, where *σ_χ _*is defined in Eq. 11. This criterion imposes that the expression profiles must be well reproduced for each cluster individually. The most reduced solution that satisfies this constraint is selected for every reduction procedure, for every model structure and for every time series. These solutions are indicated in Additional file 1: Figure S2 and their characteristics are given in Table 1.

For the embryonic time series, even the non-reduced solution obtained with the structure $m_{NC}^{e}$ fails to fulfill this more stringent criterion (*σ*_χ_≤0.5 ∀c), and therefore no reductions are indicated in the Table. The same holds for the structure $m_{CN}^{\exp}$ for the full time series. We are thus left with the two model structures $m_{CN}^{\exp}$ and $m_{NN}^{\exp}$ for the embryonic series and the two structures $m_{NC}^{\exp}$ and $m_{CN}^{\exp}$ for the full series. For each of these structures we dispose of five reduced solutions based on the procedures Ψ*_v_*, Ψ*_σ_*, $\Psi_{F}^{-}$, $\Psi_{F}^{+}$ and Ψ*_P_*. Among these, the best solutions are those that satisfy the criterion *σ*_χ_≤0.5 ∀c and have: the lowest value of the deviation between estimated and experimental profiles, *σ*; the lowest value of this deviation after perturbation of the parameters, *σ*_περτ_; the lowest value of the extrapolated expression level, χ; and the lowest number of parameters, *p*. The best solutions so selected are indicated in Table 1.

The most reduced of these best solutions have an average of two connections per node for the embryonic time series, and five connections per node for the full time series. The embryonic gene expression network is thus much sparser than the network of the full time series. This reflects the fact that the embryonic profiles are much simpler to reproduce than those of the full series. Indeed, the four development stages of the *Drosophila *show different gene expression profiles, where the transition from one stage to the next is encoded by abrupt changes [21].

**Figure 5:**
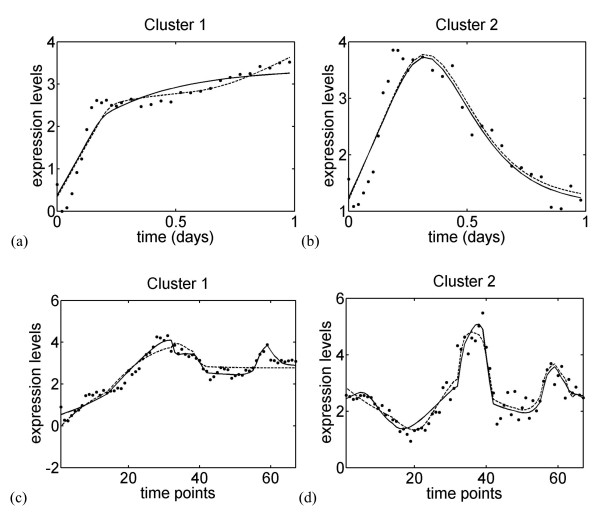
**Experimental (${\bar{X}}_{c}$) and estimated (${\hat{\bar{X}}}_{c}$) gene expression profiles for four clusters, obtained with the selected reduced solutions**. These solutions correspond to the gray lines in Tables 1, 2. Clusters 1 and 2 of the embryonic stage; dots: experimental data; solid line: model structure $m_{NN}^{\exp}$ with the reduction scheme Ψ*_σ_*; dashed line: model structure $m_{CN}^{\exp}$ with the reduction scheme Ψ*_v_*; the curves are given as a function of the real time. (c-d) Clusters 1 and 2 of the full time series; dots: experimental data; solid line: model structure $m_{NN}^{\exp}$ with the reduction scheme ΨF-; dashed line: model structure $m_{NN}^{\exp}$ with the reduction scheme Ψ*_v_*; the curves are given as a function of the time-points.

As shown in Figure 5 and Additional file 1: Figures S3-S4, our best solutions reproduce the experimental gene expression profiles very well. This is true both for the embryonic phase and the full time series, owing to the larger number of connections.

### Analysis of cluster networks

**Figure 6:**
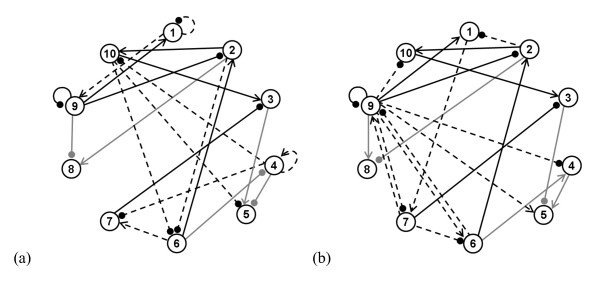
**Gene regulatory network of selected reduced solutions for the embryonic stage**. The best reduced solutions obtained with the model structures $m_{NN}^{\text{exp}}$ and $m_{CN}^{\text{exp}}$ for the embryonic stage, as defined by a good reproduction of the data, a low number of parameters, a good robustness with respect to parameter variations and a good stability in time (corresponding to the two gray lines in Table 1). The arrows and dots represent activation and inhibition, respectively. The black and gray solid lines represent the connections shared by both solutions, with the same sign or opposite sign, respectively. The connections that are present in only one of these solutions are indicated with dashed black lines (a) Network obtained with the model structure $m_{NN}^{\text{exp}}$ and the reduction scheme Ψ*_σ_*; (b) Network obtained with the model structure $m_{CN}^{\text{exp}}$ and the reduction scheme Ψ*_v_*.

The cluster regulatory networks defined by the different solutions selected in the previous section were further analyzed. For the embryonic times series these solutions are obtained with the model $m_{NN}^{\exp}$ and the parameter reduction scheme Ψ*_σ_*, and with $m_{CN}^{\exp}$ and Ψ*_v_*; for the full time series they are obtained with $m_{NN}^{\exp}$ and $\Psi_{F}^{-}$, and with $m_{NC}^{\exp}$ and Ψ*_v _*(see Tables 1, 2). The corresponding gene regulatory networks are depicted in Figures 6a-b for the embryonic time series and in Additional file 1: Figures S5a-b for the full time series. Given that the networks for the full time series have on average 5 to 7 connections ending at each node, they are quite complex and difficult to analyze further. Thus we focused on the networks for the embryonic series that have on average two connections arriving at each node.

The two networks selected for the embryonic stage are almost equally sparse, which is in agreement with the current knowledge about gene expression networks. They have moreover some connections in common. For example, cluster 3 is linked to the three clusters 6, 7 and 10 in both networks, and cluster 9 auto-represses itself. In contrast, some connections are very different, as for example those starting or ending at cluster 9, which is a hub in one of the networks and not in the other. It is impossible to determine what is the most realistic network on the basis of the available DNA microarray data alone.

A way to support our results is to compare the obtained networks with experimental data. The comparison is not straightforward, as we deal here with relations between clusters of genes that have similar expression patterns, whereas the experimental data apply to individual genes. Moreover, we look for the dynamical influences of genes on the expression rate of other genes, whereas experimental data focus on physical interactions between genes and/or gene products, coexpression of genes, or functional relationships between genes or pathways. However, there is an overlap between these different kinds of information. In particular, genes and/or gene products that interact during some or all development stages can be expected to be in the same cluster when the classification encompasses these stages. Alternatively, they can be expected to influence each others' expression rate. In contrast, two genes in the same cluster are not necessarily coexpressed, sharing a common function or involved in the same pathway. The only certainty is that they have similar expression profiles during the development stages under consideration.

To get a better biological understanding of our networks, we compare them with other predicted networks and with experimental data. The gene networks involved in the segmentation of the fly embryo have been thoroughly studied (for a review, see *e.g*. [26]) and modeled using logic-based approaches [27,28], but unfortunately most of these genes are not part of the DNA microarray time series studied here. Another well-studied subset of genes, which are all part of the time series, concerns muscle development [20]; their gene regulatory subnetwork has been predicted using a probabilistic modeling approach [29]. We focused on this subnetwork, which contains 20 genes.

We first note that our clustering procedure groups several of these 20 genes in the same cluster. More precisely, these genes are distributed among 7 clusters when considering the embryonic time series and among 5 clusters for the whole series. To be able to compare our modeled networks with the model of [29], which we will refer to as Z-network, we first translated the latter into a cluster network by defining an oriented connection between two clusters when at least one of the genes they contain shows that connection. We then compared this network with ours, and in particular with the two best non-reduced models and the two best reduced ones, which are ($m_{NN}^{\exp}$), ($m_{NN}^{\exp}$, Ψ*_σ_*), ($m_{CN}^{\exp}$) and ($m_{CN}^{\exp}$, Ψ*_v_*) for the embryonic time series (see Table 1), and ($m_{NN}^{\exp}$), ($m_{NN}^{\exp}$, $\Psi_{F}^{-}$), ($m_{NC}^{\exp}$) and ($m_{NC}^{\exp}$, Ψ*_v_*) for the full series (see Table 2). We find that these different networks share between 17% and 50% of the Z-network connections, taking the connections' orientations into account. There is thus a significant overlap between our models and the Z-model.

Another way to get insight into our results is to compare the predicted connections with physical protein-protein or protein-gene interactions. Such interactions are listed in the DroID database [30]. Among the experimentally determined interactions between a transcription factor and a gene, which are contained in this database and for which the transcription factor and the gene are in two different clusters, 38% to 69% correspond to connections present in our 8 best (abovementioned) solutions. For the experimentally determined protein-protein interactions, the correspondence is even higher: 50% to 100%. Strikingly, the correspondence between the Z-network and the experimental interactions is lower: for the transcription factor-gene interactions, the correspondence is 6% when considering individual genes, 38% when considering embryonic clusters, and 57% when considering the clusters of the full time series; for the protein-protein interactions, the values are 25%, 50% and 75%. Our results are thus encouraging and support our approach.

## Conclusions

We tested the ability of five model structures, one linear and four non-linear, to reproduce the gene expression profiles across the whole life span of *Drosophila*, or the profiles limited to the embryonic phase. The linear model *m_lin _*led to very good data reproduction, with few parameters, but turned out to be much too sensitive to parameter variations, and to yield unrealistic values of the expression levels when extrapolated in time. This model was rejected because it was incapable of absorbing the stochastic variations inherent to all biological systems and keeping the estimated values in a biologically reasonable range.

The parameter identification procedure developed here contained two steps: selection of the connections that are necessary to reproduce the data, which was achieved by minimizing *ζ *the standard deviation between the time derivatives of estimated and experimental gene expression profiles; and optimization of the parameters defining these connections by minimizing *σ *the standard deviation between the estimated and experimental gene expression profiles. Although this procedure is adequate for non-linear model structures, an easier method can be used for linear structures. It also consists of two steps. The first step consists of linear, least-squares parameter identification so as to minimize *ζ*, and the second step entails non-linear optimization of these parameters so as to minimize *σ *[9]. The existence of alternative parameter identification methods for the linear structure gave us the opportunity to test the performance of the new procedure developed here, by using both on the linear model. For the embryonic stage, we found *σ *to be equal to 0.32 with both methods. This result corroborates the validity of the present approach. Note that lower *σ*-values can be reached when considering the logarithm of the gene expression profiles ${\bar{X}}_{c}\left(\tau \right)$[9], because this function tends to smooth out the profiles.

Among the four non-linear model structures, $m_{NC}^{pol}$, which has been developed previously [11] to model a prokaryotic system subject to glucose-lactose diauxie and where the transcription term is proportional to the probabilities that the promoter is bound to an activator and not to a repressor, failed to reproduce correctly the *Drosophila *gene expression profiles and was rejected. Two reasons can be invoked to explain why this biology-based structure did not work. The first is that it has been developed for prokaryotes, where the transcriptional and translational regulation machineries are much simpler. For instance, one single repressor (activator) is able to repress (activate) gene expression in such systems, whereas in eukaryotes large biomolecular complexes are usually required. The second reason is that this transcription term is physical for gene expression networks involving single genes, but not for gene clusters. Some arguments have been presented to justify the use of this model structure for gene clusters [11], but they are based on approximations that may not be valid in the present case.

The three remaining non-linear model structures include a transcription term and/or a degradation factor that is constructed from ratios of exponential terms of the form $\exp\left(-\overset{C}{\underset{d=1}{\sum }}K_{cd}{\bar{X}}_{d}\left(t\right)\right)$. These structures, *m^exp^*, are much more flexible and encode the possibility that gene regulation is driven by biomolecular complexes. The three model structures considered differ by the constancy of the transcription term or degradation factor: $m_{CN}^{\exp}$ has a constant transcription term, $m_{NC}^{\exp}$ a constant degradation factor, and for $m_{NN}^{\exp}$ neither is constant. As $m_{NN}^{\exp}$ includes the other two models, it should in principle always outperform them. However, this is not always so, because its larger number of parameters sometimes entails identification problems. Besides, $m_{CN}^{\exp}$ does not systematically outperform $m_{CN}^{\exp}$, nor the opposite: the former is better for the embryonic stage and the latter for the full time series. But in all tested cases, at least two of the three *m^exp ^*model structures reproduced the data very well, as clearly seen in Figure 5 and Additional file 1: Figures S3-S4.

In addition to fair data reproduction, the biologically crucial properties that make the *m^exp ^*family of model structures adequate for modeling *Drosophila *gene expression across development, is their generally robust behavior against parameter variation and their large stability upon extrapolating the solutions in time. These structures are therefore selected for further analysis.

To get rid of the unnecessary parameters and connections in the *m^exp ^*model structures, several reduction procedures were defined and applied. The two simplest procedures, Ψ*_v _*and Ψ*_σ_*, where the former amounts to dropping the parameters of smallest absolute value and the latter to keeping the parameters that increase *σ *the least, gave in general the best results in terms of data reproduction, robustness against parameter perturbations and stability upon extrapolation in time. The common procedure $\Psi_{F}^{-}$, which amounts to dropping parameters that are correlated with others and are the least sensitive in the Fisher sense, *i.e*. the most robust with respect to infinitesimal parameter variations, was in general less efficient (although it gave one of the best reduced solutions). The variant $\Psi_{F}^{+}$, which drops parameters that are the most sensitive in the Fisher sense yielded similar performances. This surprising result is probably due to the fact that the most important property of the Fisher matrix-based reduction procedures is to minimize the correlation between parameters. The last reduction scheme tested, which amounts to dropping parameters that are the most sensitive to finite perturbations, usually did not allow the elimination of many parameters and thus showed the lowest performances.

We finally selected the best reduced solutions, for the embryonic stage and the full time series. These solutions turned out to have all required characteristics: good data reproduction, robustness against parameter variations, stability when extrapolating in time, and a reasonably low number of parameters. Note that parameter reduction does not have the general tendency of increasing the robustness and stability of the non-linear models (see Additional file 1: Figure S2), as it is the case for the linear models [31] (without nevertheless reaching a sufficient robustness level). These best non-linear solutions show a mean number of connections equal to two for the embryonic stage and five for the full time series. The associated networks are thus quite sparse, especially for the embryonic stage, in agreement with experimental results on *E. coli *[32]. We can thus conclude at this stage that the model structure *m^exp^*, the two-step parameter identification procedure developed here and the two reduction schemes Ψ*_v _*and Ψ*_σ_*, are all together appropriate for modeling the *Drosophila *gene expression across development.

Although overfitting of the models' parameters can never be totally excluded in the absence of thorough cross validation, our reduction procedure is designed to avoid this problem. Indeed, the original solutions, which might suffer from overfitting, are reduced until their *σ *values start to exceed a threshold value, above which the correct reproduction of the data is no longer guaranteed. The parameters of the most reduced solutions can thus be assumed not to be overfitted. Note also that the number of parameters of the reduced solutions are much smaller than the number of data points (see Tables 1, 2). For the two best reduced solutions in particular, there are 62 and 78 parameters and 310 data points in the embryonic stage, and 108 and 187 parameters and 804 data points in the full time series.

However, our results suffer from an important drawback, that is, that many gene expression networks and parameter values can be found which have approximately the same performance in terms of our different criteria, and cannot be ranked on the basis of the available data. We would like to emphasize that the biological constraints we have introduced, namely the robustness against parameter variations and the stability of the solutions upon extrapolation in time, limit the possible model structures and parameter ranges, and thus partially lift degeneracy, but not completely. Without additional data, it is impossible to determine which of the networks is the most realistic. The inclusion of other types of data and subsequent analysis of whether this renders the solution unique will be the focus of future research. Also, the application of our approach to the gene expression across the development of other organisms, or to systems subject to external perturbations such as stress, will also lead to relevant insights.

Notwithstanding the nonuniqueness of our predicted cluster networks, they compare favorably with experimental data. Indeed, focusing on the well-studied gene subset involved in muscle development, we observed that many of the partners of the experimentally identified transcription factor-gene and protein-protein interactions are members of the same gene cluster. In many other cases, the clusters these partners belong to are connected in the predicted networks. These results will be thoroughly analyzed and confirmed in further studies on the basis of experimental data on other gene subsets.

It can also be argued that the non-uniqueness of the network is actually a correct result, and can be due to the inherent plasticity of gene expression networks, where a same external perturbation can lead to different gene expression responses [33]. However, it is not obvious that such a mechanism applies to gene expression across an organism's development. As a last comment, we would like to suggest the hypothesis that many of the networks selected by our approach are valid, not because of network plasticity but because our networks connect clusters rather than single genes. These networks can thus be viewed as superimpositions of single-gene networks. If we could disentangle these networks, we would probably realize that some gene subsets are regulated according to one of the networks, and other gene subsets according to other networks. The large number of networks and solutions found for gene clusters would then be fully relevant and useful to disentangle the gene regulatory mechanisms.

## Competing interests

The authors declare that they have no competing interests.

## Authors' contributions

AH, JA and MR designed the research and analyzed the results. AH performed the modeling. MR wrote the paper. All authors read and approved the final manuscript.

## Supplementary Material

Additional file 1 **Supplementary material**. Click here for file
