# Supplementary material for: Robust non-linear differential equation models of gene expression evolution across Drosophila development
Source: BMC Res Notes. 2012 Jan 19;5:46. doi: 10.1186/1756-0500-5-46 (PMC3398324; doi:10.1186/1756-0500-5-46)
Supplement: Additional file 1 — Supplementary material. [file 1756-0500-5-46-S1.PDF]

Supplementary Material

Table S1. List of the *Drosophila* genes that are members of the different clusters. The clusters are obtained as described in section 2b.

Table S1.a Ten clusters of the embryonic stage

Class	Members
1	CG4750, CG6664, CG3666, CG2061, CG2046, CG3397, CG8950, CG4702, CG18238, CG5629, CG1691, CG10657, CG6899, CG3510, CG18218, CG1681, CG16820, CG11888, CG15288, CG1849, CG6718, CG8592, CG5568, CG7660, CG7144, CG10686, CG11183, CG16758, CG5452, CG1822, CG12892, CG2707, CG3938, CG4039, CG13316, CG11660, CG10370, CG1782, CG2145, CG15658, CG9067, CG7074, CG2902, CG15319, CG1746, CG11010, CG5058, CG7143, CG7379, CG9796, CG14999, CG3429, CG4799, CG10965, CG4224, CG9057, CG1921, CG8677, CG18437, CG11945, CG3289, CG13770, CG3658, CG15610, CG3753, CG18582, CG11329, CG11128, CG8919, CG5553, CG13397, CG7655, CG11330, CG17161, CG1655, CG7530, CG6339, CG10237, CG14712, CG8603, CG4466, CG13184, CG5263, CG6868, CG10218, CG10825, CG1453, CG1605, CG5534, CG9924, CG5370, CG9383, CG13773, CG1239, CG7780, CG7101, CG8374, CG2996, CG8727, CG4449, CG6719, CG10988, CG12024, CG10955, CG2921, CG11334, CG11971, CG1519, CG11448, CG1763, CG11347, CG17469, CG4916, CG14303, CG7340, CG6023, CG10018, CG11055, CG8826, CG5490, CG10439, CG8195, CG10444, CG1044, CG7264, CG12819, CG1489, CG12123, CG7108, CG10415, CG15084, CG14771, CG12237, CG12104, CG3800, CG12942, CG6319, CG4604, CG7745, CG2310, CG9925, CG6967, CG14459, CG4196, CG17064, CG6292, CG6542, CG12054, CG8171, CG4742, CG10622, CG1474, CG15426, CG17509, CG9890, CG9537, CG5112, CG5194, CG10293, CG7163, CG8783, CG5067, CG15435, CG5275, CG13853, CG3042, CG8735, CG2998, CG9986, CG17520, CG7628, CG7033, CG10308, CG3239, CG6678, CG5202, CG6833, CG2478, CG8156, CG5780, CG17260, CG4735, CG4993, CG7995, CG1602, CG5105, CG8155, CG1408, CG8187, CG6401, CG11490, CG12238, CG7878, CG13594, CG11844, CG10566, CG8240, CG2048, CG7843, CG9523, CG7948, CG4617, CG5184, CG9104, CG3881, CG8485, CG1416, CG10954, CG17294, CG7717, CG6453, CG17818, CG2331, CG7623, CG6465, CG10840, CG4267, CG9874, CG13671, CG5289, CG8180, CG8174, CG7015, CG10387, CG6382, CG10385, CG1236, CG6098, CG5634, CG10716, CG8669, CG4643, CG13890, CG4317, CG15312, CG3269, CG10346, CG2982, CG4265, CG7627, CG7437, CG12082, CG8572, CG3920, CG14442, CG10234, CG9670, CG18543, CG9709, CG16928, CG10209, CG9908, CG7359, CG7506, CG11290, CG12598, CG7388, CG7263, CG5797, CG5140, CG6406, CG2621, CG2943, CG8994, CG3461, CG9288, CG11208, CG11019, CG12734, CG8825, CG2698, CG10997, CG8062, CG13601, CG15151, CG17159, CG6203, CG17420, CG10602, CG1583, CG12878, CG3390, CG13343, CG6404, CG3528, CG10428, CG1963, CG7334, CG7671, CG5643, CG10171, CG11181, CG11259, CG4061, CG15104, CG8431, CG3570, CG2917, CG3041, CG10795, CG9099, CG17686, CG1201, CG3877, CG6287, CG7935, CG8548, CG7977, CG17746, CG1707, CG5776, CG1034, CG7730, CG18623, CG1848, CG4759, CG1100, CG5251, CG4626, CG18253, CG12795, CG14444, CG8084, CG17149, CG6662, CG3262, CG11207, CG6325, CG6276, CG13620, CG9581, CG18466, CG4162, CG6087, CG1794, CG17019, CG6009, CG1938, CG17370, CG10165, CG3279, CG9791, CG16805, CG7413, CG2913, CG8370, CG8947, CG14971, CG5412, CG18662, CG7178, CG7417, CG5081, CG5649, CG3359, CG2246, CG7479, CG2025, CG11194, CG2150, CG5686, CG2092, CG8402, CG17492, CG2794, CG12397, CG2856, CG4852, CG10420, CG14899, CG12316, CG3812, CG11940, CG9896, CG9054, CG1105, CG5018, CG6144, CG5378, CG6984, CG12127, CG6672, CG1532, CG5729, CG1824, CG17046, CG9071, CG7913, CG10797, CG3445, CG13605, CG1902, CG9578, CG6647, CG2970, CG9828, CG11473, CG12775, CG8261, CG10520, CG8272, CG12071, CG11344, CG4905, CG1951, CG4951, CG15086, CG11534, CG2147, CG4979, CG14969, CG7777, CG12304, CG7168, CG3618, CG9559, CG11796, CG2179, CG10873, CG8887, CG11278, CG1522, CG9536, CG10222, CG17994, CG4338, CG1146, CG8651, CG18112, CG6706, CG8857, CG9074, CG5032, CG9865, CG5916, CG6405, CG3662, CG6622, CG11186, CG8654, CG11804, CG5807, CG11316, CG9579, CG6197, CG10897, CG9119, CG5788, CG16972, CG7361, CG15618, CG10706, CG2699, CG9584, CG9201, CG3416, CG9264, CG13630, CG5914, CG6539, CG6845, CG11685, CG1287, CG8765, CG11919, CG10099, CG14461, CG4681, CG9282, CG17184, CG8329, CG12832, CG1909, CG12015, CG2604, CG10977, CG11139, CG8043, CG13604, CG9886, CG1090, CG4546, CG6605, CG10192, CG7783, CG17759, CG8576, CG16783, CG9085, CG1534, CG14032, CG4713, CG8278, CG8109, CG14472, CG8235, CG7443, CG3671, CG14183, CG7041, CG4790, CG8358, CG5931, CG12115, CG6838, CG9392, CG5641, CG17446, CG15867, CG12125, CG11869, CG2671, CG1615, CG6069, CG11428, CG11509, CG13918, CG3492, CG11908, CG17739, CG3121, CG5867, CG11404, CG8046, CG2907, CG9861, CG11475, CG9010, CG4294, CG13796, CG8136, CG17907, CG12136, CG18396, CG10097, CG17108, CG18404, CG10096, CG6372, CG18568, CG18284, CG7738, CG8813, CG17837
2	CG6670, CG7157, CG11622, CG9344, CG18374, CG9580, CG1552, CG6745, CG6564, CG10536, CG2198, CG12007, CG4889, CG15557, CG7895, CG7230, CG1133, CG5888, CG10159, CG10619, CG7902, CG2151, CG10859, CG4500, CG7757, CG12045, CG11451, CG10206, CG6234, CG7088, CG4722, CG3724, CG1374, CG17228, CG3936, CG17348, CG4999, CG13425, CG12744, CG10630, CG9140, CG11280, CG9246, CG17148, CG4389, CG7434, CG6692, CG17515, CG2163, CG4033, CG1028, CG4947, CG11440, CG8896, CG10487, CG4094, CG9885, CG5258, CG8355, CG4307, CG10595, CG3722, CG8591, CG17506, CG3303, CG6770, CG9763, CG1383, CG1721, CG8289, CG18455, CG4447, CG6988, CG1381, CG4600, CG3108, CG11782, CG10194, CG10217, CG9766, CG5695, CG5002, CG12279, CG7873, CG15427, CG6057, CG8929, CG17608,

	CG9630, CG8995, CG13384, CG4581, CG10341, CG9165, CG11212, CG7925, CG10283, CG5567, CG12638, CG12389, CG13784, CG7216, CG18350, CG3499, CG11958, CG12040, CG7509, CG15081, CG2694, CG7748, CG11763, CG11964, CG7563, CG6293, CG14768, CG4124, CG9308, CG10108, CG17752, CG6206, CG1751, CG11790, CG4842, CG5942, CG1710, CG2578, CG11063, CG10327, CG6822, CG11967, CG16761, CG4461, CG4233, CG5902, CG1888, CG5953, CG6016, CG6829, CG6501, CG6582, CG2789, CG12273, CG7148, CG5826, CG8775, CG10928, CG3297, CG7765, CG18273, CG8861, CG6860, CG16882, CG9398, CG4909, CG6687, CG3978, CG5212, CG17082, CG1553, CG1671, CG4919, CG3365, CG1233, CG3845, CG4347, CG10146, CG9663, CG10497, CG1358, CG3856, CG2226, CG8862, CG9343, CG2358, CG18426, CG5539, CG3645, CG16932, CG4538, CG12321, CG3764, CG8481, CG17309, CG2675, CG9075, CG8954, CG9543, CG7613, CG1354, CG7314, CG7967, CG5977, CG5809, CG4710, CG14205, CG5186, CG5037, CG8186, CG4608, CG9746, CG8439, CG6272, CG4768, CG8611, CG10711, CG7795, CG4769, CG9799, CG8818, CG1092, CG5995, CG17077, CG14630, CG3584, CG13458, CG7070, CG13782, CG10577, CG7364, CG9775, CG17437, CG9186, CG12096, CG3353, CG7531, CG16705, CG8251, CG4928, CG1742, CG1021, CG4491, CG2135, CG17238, CG9019, CG8230, CG5010, CG6116, CG6446, CG2275, CG8312, CG4141, CG4751, CG10473, CG18539, CG10777, CG8817, CG5707, CG17369, CG11783, CG5904, CG14125, CG5627, CG5165, CG1726, CG5670, CG11887, CG8789, CG7103, CG4336, CG3571, CG5938, CG5887, CG7656, CG10076, CG14991, CG10373, CG2835, CG6695, CG3567, CG10731, CG8026, CG5920, CG4535, CG5844, CG7065, CG3093, CG8944, CG9268, CG17822, CG13607, CG7135, CG7296, CG6313, CG4961, CG9653, CG10286, CG3172, CG9852, CG10527, CG6503, CG17274, CG1715, CG8996, CG8445, CG2765, CG7961, CG12210, CG5373, CG12156, CG5841, CG9848, CG16979, CG2641, CG10084, CG3257, CG10053, CG10254, CG1086, CG13298, CG10863, CG8460, CG7183, CG5502, CG8428, CG1371, CG2096, CG1640, CG7383, CG1893, CG7816, CG12375, CG3192, CG5889, CG6686, CG17654, CG8616, CG15012, CG12167, CG5163, CG11148, CG11407, CG4823, CG5073, CG18176, CG1537, CG3327, CG12168, CG14045, CG4432, CG6923, CG3314, CG11546, CG3143, CG1559, CG4088, CG4221, CG9438, CG10383, CG5946, CG6369, CG4644, CG6699, CG5429, CG4035, CG4585, CG1099, CG1810, CG7649, CG7987, CG9063, CG1980, CG11866, CG3423, CG2006, CG6725, CG11807, CG17565, CG6811, CG5675, CG8024, CG1088, CG11309, CG3127, CG14235, CG8536, CG12251, CG5805, CG1135, CG5053, CG18525, CG5215, CG5821, CG1065, CG4592, CG12118, CG6230, CG4407, CG15099, CG3530, CG3036, CG10967, CG5562, CG1017, CG4144, CG5859, CG5374, CG3182, CG9485, CG9749, CG14296, CG8092, CG6043, CG14290, CG1009, CG6903, CG7220, CG6283, CG2190, CG8613, CG18627, CG3752, CG6042, CG3731, CG8416, CG15437, CG3810, CG3173, CG13925, CG2171, CG17272, CG13812, CG13598, CG5741, CG11883, CG3441, CG17514, CG9131, CG3992, CG5815, CG5555, CG1891, CG12212, CG17332, CG6467, CG7099, CG4214, CG4186, CG8286, CG9934, CG5119, CG3857, CG11074, CG6734, CG12348, CG14718, CG3663, CG1241, CG2385, CG11656, CG11880, CG11963, CG9317, CG12182, CG8549, CG14721, CG4288, CG8415, CG3200, CG7441, CG8298, CG8135, CG17841, CG1107, CG8593, CG6367, CG1874, CG2077, CG6128, CG6371, CG12232, CG4782, CG17597, CG8182, CG9554, CG9244, CG4663, CG7007, CG12163, CG14318, CG7829, CG9507, CG9176, CG4266, CG12233, CG4955, CG7672, CG12770, CG5325, CG8806, CG3253, CG2723, CG6661, CG8565, CG18494, CG14942, CG8620, CG9668, CG9136, CG15309, CG9483, CG7090, CG16727, CG10672, CG7886, CG8012, CG11841, CG5150, CG7342, CG7557, CG3573, CG2088, CG5735, CG2750, CG17645, CG17838, CG1979, CG10934, CG2209, CG11703, CG2854, CG10752, CG3557, CG17210, CG4836, CG7634, CG5576, CG10841, CG1744, CG1340, CG4715, CG4714, CG4323, CG10888, CG17012, CG1324, CG17349, CG3565, CG7251, CG7077, CG6541, CG15208, CG8701, CG4784, CG8023, CG14735, CG13030, CG12374, CG2149, CG2127
3	CG4550, CG13124, CG5758, CG8505, CG17884, CG1803, CG11914, CG3714, CG2528, CG4766, CG10596, CG7107, CG1441, CG13868, CG3961, CG11142, CG15131, CG12466, CG3364, CG17248, CG3421, CG3238, CG5973, CG7981, CG6981, CG7850, CG11916, CG8888, CG4639, CG1124, CG17927, CG9633, CG12052, CG7321, CG2858, CG8855, CG3820, CG5326, CG5393, CG12449, CG15560, CG18178, CG12214, CG1395, CG3795, CG6028, CG3006, CG2727, CG9568, CG18285, CG10953, CG1765, CG6105, CG8553, CG1780, CG4302, CG14996, CG14682, CG4123, CG8049, CG15855, CG9489, CG14896, CG5596, CG4827, CG8602, CG5772, CG7055, CG11961, CG8247, CG5468, CG13480, CG11417, CG4879, CG9336, CG4747, CG5584, CG14989, CG3588, CG12756, CG11387, CG13089, CG4533, CG9293, CG6625, CG5589, CG15165, CG4122, CG7028, CG2803, CG10719, CG9723, CG5383, CG5279, CG5095, CG10026, CG2244, CG7896, CG8154, CG5875, CG10121, CG18512, CG1071, CG9338, CG6701, CG1070, CG11390, CG10043, CG15444, CG9619, CG4037, CG2467, CG2926, CG1057, CG7993, CG4528, CG11130, CG9467, CG10814, CG3480, CG12846, CG9780, CG16858, CG2507, CG17958, CG3941, CG6474, CG11837, CG9443, CG4526, CG7614, CG7764, CG14211, CG14233, CG14528, CG10939, CG3878, CG7470, CG3413, CG8367, CG9166, CG4427, CG4375, CG6509, CG4402, CG9356, CG2155, CG4677, CG17689, CG3987, CG14216, CG12749, CG11910, CG15804, CG9701, CG3902, CG6394, CG5055, CG3823, CG4120, CG1479, CG1228, CG18551, CG9972, CG3762, CG10311, CG8399, CG17836, CG6794, CG5965, CG7724, CG3594, CG18033, CG8327, CG7011, CG11275, CG16724, CG7708, CG3427, CG7480, CG1944, CG10693, CG5411, CG1106, CG9021, CG15900, CG5135, CG1809, CG10641, CG10143, CG8846, CG5877, CG12840, CG1163, CG4260, CG10521, CG6166, CG9088, CG1591, CG9331, CG4164, CG7600, CG8250, CG2952, CG11504, CG1516, CG1600, CG5308, CG9164, CG3991, CG4654, CG7400, CG16936, CG8372, CG11960, CG9335, CG17156, CG7142, CG14724, CG11466, CG1512, CG8854, CG17493, CG6906, CG6667, CG8032, CG9480, CG9321, CG11354, CG3779, CG11712, CG3415, CG13852, CG7704, CG9208, CG12130, CG17888, CG8864, CG12606, CG11426, CG5237, CG9437, CG3161, CG6305, CG13432, CG5813, CG7939, CG3998, CG9312, CG8511, CG11170, CG18638, CG5939, CG7846, CG1826, CG11779, CG7456, CG11586, CG10627, CG17875, CG8232, CG16719, CG4069, CG8242, CG18061, CG12781, CG5871, CG12403, CG6754, CG3937, CG6844, CG6593, CG12134, CG13626, CG5687, CG5845, CG9947, CG3533, CG6496, CG2736, CG6315, CG5288, CG7149, CG1648, CG14646, CG17617, CG9512, CG5594, CG4232, CG3973, CG18093, CG2930, CG1103, CG12004, CG9889, CG18409, CG4168, CG17111, CG2505, CG10106, CG4918, CG3446, CG3324, CG6942, CG7565, CG4019, CG11073, CG18104, CG7093, CG4891, CG3020, CG10384, CG6958, CG7525, CG1607, CG6740, CG11274, CG6742, CG9008, CG15279,

	CG14830, CG11711, CG6006, CG8462, CG8279, CG5684, CG5407, CG4826, CG12129, CG10347, CG17397, CG13800, CG12173, CG12612, CG16926, CG9400, CG10204, CG8766, CG18444, CG6092, CG18214, CG1970, CG15613, CG7720, CG9306, CG8381, CG6282, CG10142, CG6939, CG13830, CG5793, CG10120, CG11625, CG14207, CG9674, CG10039, CG3308, CG7231, CG9932, CG6773, CG5282, CG7940, CG15688, CG12295, CG8086, CG8444, CG5059, CG10947, CG8909, CG9836, CG5291, CG6058, CG6684, CG10675, CG3318, CG6656, CG18314, CG5903, CG10365, CG17999, CG4645, CG2082, CG1208, CG17800, CG2131, CG3999, CG18596, CG5449, CG1104, CG18403, CG15811, CG9603, CG7920, CG4894, CG12268, CG10949, CG14904, CG4653, CG11191, CG15668, CG9155, CG8422, CG17336, CG17762, CG5945, CG9879, CG10131, CG10639, CG3529, CG4692, CG3616, CG12170, CG18609, CG2055, CG3241, CG5461, CG4789, CG2176, CG17027, CG17034, CG15118, CG1889, CG10126, CG3560, CG3139, CG3835, CG5827, CG11236, CG11737, CG15068, CG11462, CG5154, CG12955, CG10553, CG10664, CG12089, CG3138, CG10946, CG9151, CG9547, CG18324, CG9169, CG5071, CG3632, CG9441, CG9813, CG12473, CG10570, CG3372, CG5482, CG18138, CG4839, CG13718, CG3832, CG2677, CG11455, CG12239, CG9354, CG7105, CG6771, CG14024, CG5588, CG10985, CG7430, CG7438, CG3209, CG11221, CG6273, CG6020, CG11661, CG6123, CG12800, CG7580, CG2017, CG7050, CG7576, CG10550, CG4462, CG13240, CG3135, CG6652, CG8532, CG6335, CG15520, CG12120, CG5703, CG9122, CG7068, CG3459, CG3159, CG6432, CG15509, CG1954, CG10680, CG17926, CG9245, CG14008, CG12191, CG15360, CG7905, CG5172, CG1521, CG8782, CG9808, CG3116, CG7727, CG5712, CG10249, CG17450, CG12907, CG7635, CG7742, CG18179, CG4250, CG7915, CG7084, CG6866, CG8740, CG6456, CG10999, CG8790, CG6821, CG5089, CG8772, CG5711, CG11650, CG5571, CG8403, CG7397, CG4795, CG4468, CG7106, CG9284
4	CG5962, CG8838, CG1200, CG4899, CG8343, CG11848, CG9469, CG17052, CG7414, CG5431, CG4000, CG9411, CG11266, CG12085, CG4082, CG4354, CG8606, CG6620, CG10561, CG10230, CG10374, CG5175, CG8975, CG9839, CG11526, CG9776, CG10800, CG11210, CG10364, CG7538, CG12006, CG12869, CG3085, CG4274, CG9734, CG13848, CG7433, CG4199, CG17081, CG11403, CG1578, CG14709, CG10391, CG2050, CG4609, CG4829, CG12369, CG1866, CG4455, CG4602, CG6173, CG7788, CG4206, CG7022, CG3566, CG6015, CG18476, CG1927, CG7528, CG3221, CG5575, CG3962, CG18622, CG10355, CG5784, CG1911, CG6962, CG13849, CG14941, CG7664, CG12230, CG6791, CG8625, CG10712, CG3689, CG2199, CG2040, CG1142, CG6994, CG10849, CG4817, CG8443, CG3864, CG5868, CG9107, CG17947, CG11267, CG18362, CG4694, CG10922, CG5786, CG10390, CG9573, CG11990, CG17203, CG6746, CG6385, CG6169, CG4201, CG7845, CG1263, CG11935, CG1913, CG4700, CG11988, CG18398, CG7581, CG10151, CG17292, CG14732, CG10267, CG6418, CG5522, CG17602, CG5109, CG7892, CG9139, CG12138, CG10016, CG9300, CG8108, CG5694, CG12702, CG10851, CG14981, CG8326, CG10689, CG9535, CG6724, CG6342, CG18022, CG11276, CG3178, CG9018, CG9028, CG1528, CG9277, CG5320, CG6022, CG4621, CG5406, CG7511, CG10754, CG2218, CG1225, CG12759, CG8013, CG1918, CG9086, CG7293, CG10080, CG5290, CG15532, CG14895, CG15228, CG8833, CG7004, CG3723, CG15439, CG7102, CG8892, CG7626, CG17912, CG9680, CG3770, CG1401, CG4384, CG5787, CG3715, CG1814, CG4619, CG7338, CG10691, CG12340, CG8151, CG8621, CG10509, CG8079, CG9304, CG2031, CG6987, CG9805, CG9198, CG3696, CG2054, CG8280, CG14657, CG3129, CG15101, CG9188, CG15010, CG1582, CG9012, CG3018, CG4184, CG4548, CG6502, CG4806, CG8819, CG13627, CG15013, CG9403, CG9135, CG6884, CG5591, CG12152, CG3790, CG17170, CG9933, CG5640, CG14939, CG10555, CG3955, CG3967, CG7752, CG11700, CG5444, CG8722, CG2718, CG3075, CG4571, CG13388, CG17765, CG4152, CG9305, CG1657, CG5803, CG7549, CG12301, CG5102, CG5126, CG17712, CG5456, CG12202, CG8631, CG10123, CG8732, CG5899, CG9707, CG6841, CG10161, CG9696, CG4813, CG4563, CG8288, CG4108, CG6815, CG14722, CG9209, CG10538, CG5206, CG11547, CG5193, CG10881, CG6850, CG10869, CG4263, CG12701, CG6963, CG10671, CG17596, CG2097, CG4008, CG1444, CG3971, CG18380, CG11678, CG9373, CG8946, CG5926, CG17035, CG9878, CG18124, CG3727, CG7188, CG18593, CG17367, CG4659, CG10065, CG9031, CG1635, CG6905, CG14213, CG7803, CG8153, CG10701, CG6584, CG3203, CG15608, CG4364, CG1846, CG12075, CG5822, CG8083, CG10210, CG14178, CG9337, CG8531, CG6922, CG12050, CG3702, CG7415, CG4699, CG4972, CG7392, CG10376, CG7057, CG16973, CG6375, CG5486, CG6758, CG1658, CG11206, CG6523, CG10274, CG8465, CG15445, CG2976, CG18107, CG2182, CG8877, CG12113, CG7515, CG4118, CG17998, CG11877, CG5523, CG18011, CG6603, CG10923, CG1818, CG4763, CG7391, CG2144, CG4716, CG12211, CG8201, CG2934, CG5919, CG10144, CG1646, CG7859, CG1737, CG7946, CG10590, CG7483, CG14231, CG1108, CG6359, CG14777, CG4446, CG15667, CG7962, CG3603, CG11293, CG4974, CG4376, CG7158, CG1898, CG7147, CG7564, CG4612, CG3885, CG18497, CG4925, CG2168, CG5455, CG9797, CG18212, CG8793, CG1787, CG6995, CG5469, CG9779, CG4878, CG5835, CG9172, CG7776, CG11352, CG9591, CG6424, CG4975, CG1965, CG17342, CG8981, CG11738, CG12079, CG10409, CG13323, CG5725, CG1862, CG16982, CG2469, CG10966, CG11970, CG7058, CG1216, CG3291, CG7177, CG12355, CG6851, CG16989, CG7897, CG17090, CG11427, CG5742, CG4013, CG3411, CG8401, CG10755, CG9577, CG7162, CG16701, CG8902, CG14228, CG8206, CG8580, CG1770, CG9391, CG9531, CG1081, CG14490, CG1964, CG2107, CG3793, CG1116, CG3682, CG8057, CG4564, CG14408, CG2712, CG3664, CG15386, CG11595, CG7029, CG5532, CG4966, CG8993, CG8483, CG6495, CG12184, CG1915, CG10291, CG5009, CG9323, CG8643, CG11299, CG13907, CG5004, CG9921, CG5433, CG13483, CG3612, CG12283, CG8314, CG1059, CG10155, CG3668, CG7899, CG12235, CG8060, CG8739, CG4900, CG8175, CG2045, CG4963, CG5714, CG5358, CG10366, CG7100, CG3558, CG18000, CG11955, CG13865, CG3187, CG16941, CG4810, CG8509, CG9712, CG6180, CG7794, CG1314, CG16707, CG10750, CG1760, CG7387, CG2616, CG3290, CG17302, CG3306, CG4983, CG11842, CG17470, CG10138, CG1495, CG5778, CG7096, CG7131, CG4679, CG6649, CG4913, CG6790, CG3092, CG7366, CG6255, CG9898, CG5125, CG13280, CG7815, CG6639
5	CG5397, CG8721, CG6908, CG9090, CG9686, CG10342, CG10530, CG1438, CG7953, CG13095, CG1743, CG4229, CG17903, CG3481, CG6544, CG17549, CG15093, CG8380, CG8342, CG1252, CG10475, CG10472, CG17977, CG10245, CG3541, CG5853, CG9432, CG18020, CG10248, CG9098, CG7229, CG7874, CG5001, CG14455, CG3344, CG3106, CG4962, CG10200, CG4791, CG10433, CG6933, CG10830, CG18076, CG9363,

	CG6643, CG5839, CG10241, CG6126, CG11605, CG17814, CG1623, CG12142, CG17534, CG4475, CG8774, CG7290, CG6416, CG2140, CG1865, CG2196, CG8907, CG9509, CG9390, CG8560, CG9379, CG4321, CG1545, CG7021, CG18065, CG4859, CG8547, CG4370, CG9364, CG4355, CG8167, CG10226, CG3672, CG10562, CG9510, CG5783, CG18358, CG6214, CG9675, CG8785, CG9538, CG4682, CG10833, CG16887, CG1774, CG8128, CG14704, CG11317, CG8736, CG3332, CG10806, CG6440, CG14438, CG8588, CG5740, CG5506, CG6484, CG6702, CG10842, CG12105, CG6014, CG18522, CG10116, CG5577, CG6483, CG1168, CG9466, CG2070, CG9759, CG7567, CG17137, CG5597, CG6264, CG7252, CG1121, CG9572, CG5770, CG8256, CG9665, CG14994, CG9519, CG3690, CG2225, CG2555, CG2680, CG8773, CG3331, CG1468, CG9704, CG16987, CG12240, CG10912, CG4377, CG7002, CG2081, CG3168, CG6296, CG9431, CG3301, CG5955, CG4847, CG12505, CG11470, CG11912, CG13356, CG9682
6	CG4001, CG13787, CG9195, CG1303, CG2657, CG1535, CG6455, CG10372, CG9741, CG9506, CG7045, CG8988, CG11005, CG18462, CG11035, CG4057, CG8468, CG16757, CG5261, CG3024, CG1218, CG1624, CG6425, CG2204, CG8352, -CG7524, CG5701, CG2079, CG1435, CG5940, CG8684, CG9216, CG5100, CG5632, CG8507, CG18683, CG1800, CG5971, CG1454, CG4200, CG5581, CG12244, CG6157, CG8928, CG17018, CG11771, CG3208, CG5785, CG6977, CG5203, CG9241, CG10107, CG10295, CG8486, CG8961, CG2087, CG4281, CG8390, CG12109, CG6251, CG5848, CG9126, CG7146, CG8912, CG17678, CG6665, CG15737, CG11856, CG2864, CG5880, CG5541, CG3995, CG8068, CG6258, CG4502, CG3021, CG10139, CG3273, CG5026, CG4357, CG3157, CG1896, CG7945, CG8435, CG1962, CG14749, CG7663, CG10038, CG6219, CG5639, CG4920, CG7825, CG9646, CG11560, CG3497, CG7869, CG11596, CG10255, CG6814, CG14222, CG11388, CG4656, CG7217, CG10033, CG10640, CG1091, CG3870, CG3508, CG8116, CG14889, CG9705, CG10193, CG5676, CG7081, CG12117, CG7238, CG4949, CG9027, CG17138, CG4497, CG14224, CG7824, CG4973, CG4257, CG15835, CG9953, CG12276, CG7979, CG14764, CG15893, CG9821, CG13319, CG15486, CG6147, CG7066, CG6549, CG6673, CG3399, CG2829, CG5802, CG5726, CG6407, CG4488, CG2670, CG10938, CG3654, CG7206, CG1603, CG9858, CG11484, CG10702, CG1109, CG8924, CG2947, CG5720, CG14804, CG7197, CG4390, CG4032, CG12317, CG2051, CG4166, CG16868, CG4217, CG17540, CG13840, CG6311, CG14005, CG6896, CG3561, CG2899, CG7212, CG5345, CG6179, CG7725, CG3045, CG2678, CG6674, CG4300, CG12008, CG7805, CG2910, CG12132, CG2221, CG4365, CG10961, CG12081, CG3249, CG4050, CG3167, CG3082, CG7133, CG3191, CG1643, CG8389, CG8001, CG18177, CG18042, CG9062, CG4785, CG11980, CG18533, CG3066, CG6005, CG10563, CG14100, CG1227, CG10009, CG12363, CG7319, CG3184, CG7872, CG9239, CG11367, CG3876, CG3620, CG6192, CG18041, CG18145, CG9238, CG7185, CG3281, CG3004, CG5014, CG9834, CG4618, CG5991, CG1503, CG18004, CG8983, CG1836, CG9415, CG4867, CG17556, CG8347, CG7291, CG5222, CG6961, CG17033, CG5353, CG11242, CG8284, CG7597, CG1487, CG10609, CG12737, CG2903, CG8440, CG15112, CG1868, CG2264, CG9007, CG3328, CG6345, CG5508, CG1976, CG6222, CG6422, CG8290, CG1490, CG7536, CG8185, CG9949, CG1659, CG5099, CG16798, CG8287, CG13777, CG4875, CG8433, CG6543, CG9042, CG1636, CG4865, CG3102, CG5884, CG8073, CG10978, CG11860, CG9418, CG9425, CG5608, CG2843, CG7347, CG8229, CG3309, CG4572, CG2258, CG7407, CG9351, CG8357, CG7494, CG5850, CG7853, CG7974, CG3086, CG8301, CG6550, CG4877, CG1469, CG5915, CG9319, CG12428, CG14967, CG17299, CG9231, CG8725, CG15817, CG17223, CG9446, CG1844, CG7546, CG8253, CG1341, CG1739, CG3329, CG18278, CG11715, CG1716, CG1388, CG10149, CG1258, CG17322, CG8632, CG5284, CG13219, CG3060, CG18495, CG4586, CG4316, CG5174, CG1515, CG7773, CG8067, CG2076, CG8379, CG13421, CG6567, CG5590, CG4764, CG17352, CG1539, CG17010, CG10408, CG4968, CG16772, CG7955
7	CG15848, CG3088, CG6968, CG6306, CG17736, CG1472, CG15634, CG9528, CG6439, CG6332, CG7675, CG5538, CG12699, CG4261, CG1378, CG10154, CG1394, CG1161, CG2267, CG12011, CG8031, CG18134, CG12703, CG6433, CG5779, CG6772, CG6836, CG8596, CG10591, CG9520, CG12284, CG7507, CG8960, CG9795, CG15009, CG1773, CG17835, CG16745, CG16833, CG3375, CG5651, CG14548, CG5656, CG5173, CG17843, CG13521, CG8306, CG11100, CG7802, CG13279, CG6199, CG2530, CG14954, CG17632, CG18111, CG10236, CG10621, CG4429, CG5886, CG12177, CG9089, CG14808, CG3924, CG3851, CG14025, CG5123, CG10077, CG3009, CG10512, CG1429, CG7266, CG15390, CG6716, CG12088, CG10211, CG9342, CG8189, CG4424, CG9334, CG6170, CG7176, CG9148, CG8759, CG4822, CG8600, CG5201, CG8568, CG2669, CG14766, CG7123, CG3652, CG18319, CG4482, CG17667, CG8503, CG7203, CG11353, CG11836, CG16791, CG8660, CG8633, CG12918, CG11567, CG5145, CG4668, CG6398, CG3322, CG14886, CG7611, CG7801, CG2674, CG2062, CG4719, CG12076, CG12091, CG9708, CG8639, CG1637, CG11089, CG3879, CG1942, CG14026, CG10484, CG5837, CG10382, CG1969, CG17255, CG2867, CG1977, CG13221, CG1326, CG8054, CG3619, CG11064, CG12022, CG17452, CG3333, CG6936, CG14433, CG6236, CG3376, CG7821, CG17771, CG6193, CG7097, CG17603, CG3625, CG1524, CG11045, CG5170, CG4262, CG13850, CG1791, CG1074, CG2060, CG7958, CG7518, CG6323, CG7908, CG10416, CG8144, CG5168, CG13185, CG3271, CG15095, CG1114, CG8707, CG12373, CG12758, CG1523, CG1089, CG3875, CG10733, CG11654, CG17682, CG10658, CG7469, CG9576, CG5262, CG8121, CG5164, CG12706, CG8668, CG13398, CG12894, CG10898, CG1856, CG12253, CG10545, CG10593, CG4761, CG11658, CG10238, CG11247, CG3460, CG10392, CG8552, CG10130, CG12101, CG7122, CG12207, CG3368, CG3338, CG4649, CG1451, CG10739, CG15340, CG1007, CG7586, CG1342, CG7503, CG8676, CG2103, CG8583, CG2971, CG17611, CG13551, CG1349, CG2663, CG1514, CG1309, CG8815, CG4720, CG1901, CG11454, CG7069, CG12372, CG2522, CG10724, CG8205, CG15100, CG8714, CG4898, CG3409, CG8470, CG11188, CG6714, CG6156, CG11255, CG3059, CG2162, CG17723, CG3564, CG17921, CG3287, CG11033, CG4036, CG2139, CG8494, CG3988, CG13728, CG6645, CG9968, CG2708, CG6038, CG15002, CG16778, CG12263, CG6215, CG7956, CG12204, CG7048, CG14813, CG8980, CG18019, CG3590, CG7642, CG11425, CG9715, CG9699, CG4746, CG5108, CG14080, CG12467, CG7830, CG9484, CG14938, CG8199, CG11169, CG5394, CG18546, CG5248, CG9005, CG4495, CG10289, CG16817, CG9009, CG12896, CG3304, CG5013, CG5196, CG6343, CG4501, CG9026, CG6302, CG2994, CG1903, CG1597, CG11642, CG1587, CG8266, CG10462, CG5036, CG9047, CG8243, CG3282, CG9318, CG12529, CG10846,

	CG3348, CG15814, CG17711, CG9629, CG8420, CG16959, CG3268, CG4872, CG17485, CG6912, CG6941, CG3756, CG10325, CG8190, CG1055, CG13917, CG1883, CG3156, CG9660, CG8348, CG10072, CG5721, CG8351, CG1799, CG6615, CG1806, CG12189, CG2681, CG3054, CG1651, CG4527, CG1952, CG7502, CG6388, CG1815, CG8159, CG1449, CG3758, CG11482, CG3811, CG11537, CG14689, CG7701, CG8451, CG4426, CG7841, CG10632, CG14782, CG9396, CG5382, CG14992, CG2918, CG11525, CG17221, CG5044, CG9325, CG8609, CG9958, CG10441, CG5748, CG3959, CG17809, CG12213, CG5846, CG8212, CG11061, CG3403, CG11232, CG7129, CG12099, CG4848, CG4062, CG7161, CG9769, CG11992, CG1049, CG5530, CG6757, CG3644, CG8733, CG6355, CG1322, CG7125, CG4084, CG8110, CG16944, CG17806, CG3922, CG7590, CG6779, CG17060, CG1363, CG2177, CG1210, CG10228, CG11798, CG2716, CG6285, CG10082, CG6094, CG17544, CG13130, CG11325, CG1407, CG1193, CG15513, CG7082, CG12131, CG6775, CG4685, CG7800, CG15609, CG10810, CG8453, CG5190, CG7448, CG9798, CG4078, CG12220, CG11968, CG9091, CG17952, CG12753, CG10539, CG12002, CG4525, CG12876, CG9916, CG4275, CG14997, CG17521, CG12305, CG5706, CG6117, CG6606, CG3874, CG10944, CG13330, CG5127, CG5961, CG8045, CG8705, CG15440, CG12124, CG1467, CG2818, CG17484, CG3989, CG10948, CG3195, CG8615, CG5047, CG3738, CG13603, CG10186, CG7510, CG3140, CG7654, CG8056, CG8118, CG8376, CG18352, CG9985, CG3744, CG4428, CG18459, CG2448, CG4841, CG7806, CG5191, CG17840, CG2143, CG17259, CG14792, CG11791, CG8974, CG10605, CG11371, CG4114, CG10816, CG2534, CG16742, CG3751, CG8139, CG15433, CG2984, CG6224, CG2095, CG13647, CG17894, CG2216, CG11098, CG14802, CG7702, CG18282, CG6290, CG7734, CG5840, CG14940, CG10652, CG1506, CG6145, CG10492, CG7814, CG4104, CG4276, CG4760, CG18316, CG2961, CG8237, CG17737, CG1973, CG11770, CG7277, CG11297, CG1102, CG6141, CG6233, CG3556, CG14209, CG9144, CG7484, CG5466, CG1937, CG5417, CG3160, CG1115, CG4030, CG1140, CG8409, CG10091, CG8637, CG4182, CG13503, CG6450, CG11806, CG2049, CG5231, CG14214, CG8384, CG7499, CG5287, CG17508, CG8777, CG8605, CG13714, CG15105, CG10021, CG4012, CG17524, CG7365, CG4897, CG5497, CG4662, CG10199, CG17676, CG12582, CG11874, CG4006, CG7865, CG10732, CG15772, CG3743, CG12740, CG7490, CG4422, CG6044, CG4211, CG8215, CG7111, CG6033, CG5166, CG12021, CG12139, CG15217, CG5989, CG12090, CG13889, CG3350, CG4994, CG8525, CG14195, CG3493, CG2102, CG6676, CG14723, CG4629, CG7927, CG8601, CG1041, CG8979, CG10579, CG12016, CG16716, CG3270, CG18677, CG11303, CG10128, CG1500, CG8368, CG4126, CG11248, CG4589, CG7283, CG8969, CG4703, CG9884, CG6045, CG4416, CG8715, CG8231, CG8179, CG4071, CG5966, CG6980, CG7690, CG4067, CG1464, CG8332, CG11171, CG3033, CG3982, CG10508, CG4070, CG4392, CG11999, CG6510, CG2304, CG11892, CG8814, CG8743, CG3906, CG1830, CG9717, CG4542, CG1839, CG10443, CG1412, CG9345, CG9553, CG8059, CG6528, CG15442, CG3330, CG8495, CG8657, CG2485, CG3523, CG6735, CG11414, CG4729, CG13387, CG14800, CG11591, CG17975, CG9377, CG10060, CG7887, CG4285, CG3950, CG1345, CG18317, CG8216, CG12532, CG6159, CG6458, CG4523, CG11282, CG3868, CG4268, CG9313, CG7722, CG10494, CG1277, CG1497, CG11477, CG5862, CG6668, CG1112, CG4472, CG12178, CG7834, CG18102, CG2086, CG9804, CG10860, CG4087, CG9672, CG9162, CG7529, CG2175, CG3615, CG17025, CG6331, CG1827, CG4556, CG7358, CG14617, CG18174, CG5048, CG18143, CG9056, CG4420, CG12106, CG1730, CG9448, CG9762, CG8318, CG12727, CG8884, CG7766, CG10882, CG3825, CG1905, CG4995, CG10513, CG3356, CG1212, CG7985, CG10589, CG3335, CG14786, CG12290, CG3213, CG18550, CG10757, CG10115, CG7224, CG11146, CG3126, CG10277, CG5405, CG6265, CG15891, CG9772, CG1462, CG10631, CG12982, CG15387, CG8320, CG18616, CG5404, CG6640, CG8577, CG10279, CG4353, CG1317, CG10537, CG11094, CG13049, CG18858, CG4837, CG8362, CG18255, CG3091, CG1274, CG5348, CG3504, CG6614, CG5495, CG10042, CG10916, CG12162, CG3829, CG10854, CG4557, CG1220, CG6204, CG3772, CG14935, CG3544, CG2852, CG5080, CG17769, CG6761, CG13841, CG1213, CG2457, CG3234, CG2819, CG12236, CG4059, CG8479, CG7324, CG4456, CG3074, CG4524, CG5084, CG3748, CG16844, CG8257, CG9451, CG9803, CG3483, CG8918, CG6788, CG6059, CG6707, CG18490, CG12789, CG2985, CG8866, CG17754, CG4180, CG8475, CG8345, CG9649, CG2056, CG8619, CG11120, CG4471, CG3124, CG2287, CG2233, CG1670, CG3380, CG3648, CG2830, CG10233, CG15892, CG12763, CG6910, CG9497, CG5450, CG3245, CG9137, CG7669, CG18418, CG14208, CG17564, CG12423, CG6518, CG14740, CG5983, CG4405, CG6186, CG11853, CG13223, CG6441, CG12605, CG12558, CG1835, CG6470, CG5443, CG9314, CG11459, CG7560, CG3809, CG6730, CG7906, CG1540, CG4669, CG5398, CG7607, CG10514, CG4757, CG4818, CG18337, CG1733, CG3966, CG14891, CG8040, CG11400, CG6130, CG9130, CG17022, CG8508, CG17567, CG5107, CG10202, CG5755, CG7924, CG7160, CG10742, CG7445, CG12860, CG1999, CG9920, CG9133
8	CG7159, CG14355, CG5205, CG4945, CG2330, CG2685, CG5428, CG2666, CG1153, CG6959, CG4386, CG7399, CG9689, CG7540, CG9761, CG2767, CG7240, CG7227, CG6831, CG15818, CG6611, CG4311, CG4929, CG6608, CG7571, CG6448, CG14021, CG8196, CG3767, CG10585, CG2016, CG5677, CG9505, CG10704, CG18249, CG1554, CG7300, CG8501, CG3608, CG12311, CG7486, CG17646, CG3097, CG11200, CG4678, CG18140, CG4778, CG4664, CG8363, CG9445, CG11823, CG8920, CG6572, CG9453, CG8683, CG9456, CG14995
9	CG9812, CG3315, CG1754, CG3223, CG5505, CG4622, CG8122, CG4857, CG4453, CG7999, CG7983, CG2916, CG7425, CG13340, CG7737, CG5363, CG4454, CG8931, CG5519, CG3455, CG13096, CG5692, CG2186, CG8882, CG2328, CG14965, CG1943, CG9666, CG7421, CG12306, CG5462, CG5198, CG7838, CG14226, CG7269, CG2956, CG5924, CG8295, CG9362, CG1240, CG2091, CG17129, CG10990, CG1745, CG5094, CG9450, CG12035, CG10417, CG10212, CG7728, CG16901, CG10837, CG1925, CG7583, CG4831, CG8114, CG13201, CG1536, CG6538, CG8149, CG9754, CG14641, CG1404, CG1832, CG3910, CG3735, CG5930, CG7275, CG5654, CG10203, CG7351, CG9768, CG7926, CG2691, CG3342, CG12234, CG3918, CG11993, CG3511, CG8856, CG8441, CG2446, CG13832, CG3285, CG6391, CG4510, CG6187, CG1078, CG11154, CG17273, CG8749, CG6854, CG6316, CG11820, CG3710, CG1884, CG7467, CG11050, CG10528, CG7831, CG7288, CG4985, CG8448, CG13667, CG11397, CG16753, CG2034, CG17454, CG3613, CG6210, CG11943, CG9984, CG9213, CG3428, CG3704, CG2905, CG5448, CG5422, CG2682, CG12750, CG7986, CG12179, CG11375, CG2968, CG7062, CG5580, CG7839, CG2922, CG7357, CG7682, CG11104, CG1542, CG6054,

	CG4258, CG17358, CG5954, CG1024, CG3889, CG1753, CG5355, CG6226, CG8617, CG9242, CG2790, CG1677, CG6723, CG6819, CG2890, CG17938, CG4303, CG2260, CG14683, CG10281, CG1906, CG9423, CG16892, CG6677, CG6241, CG2925, CG11723, CG2945, CG9159, CG10057, CG15694, CG11228, CG8575, CG2950, CG4943, CG3312, CG7281, CG6506, CG2503, CG4236, CG9967, CG1101, CG7832, CG1420, CG1676, CG13929, CG7558, CG8569, CG1471, CG6050, CG3847, CG3522, CG10565, CG6556, CG18608, CG17681, CG2028, CG12135, CG3180, CG8309, CG5146, CG17436, CG11808, CG3152, CG7262, CG6395, CG17153, CG11486, CG1275, CG9556, CG1647, CG11982, CG6563, CG6575, CG1596, CG4107, CG6254, CG9764, CG15512, CG2493, CG1307, CG18592, CG5510, CG9177, CG3305, CG3458, CG3073, CG8233, CG12030, CG11471, CG3628, CG16812, CG2158, CG7199, CG3400, CG14650, CG5808, CG1530, CG1708, CG7098, CG13097, CG7705, CG8571, CG11897, CG7073, CG6363, CG4192, CG2831, CG16788, CG8949, CG11456, CG2862, CG3726, CG6238, CG8378, CG7109, CG6911, CG7012, CG3887, CG12121, CG3634, CG4577, CG7049, CG11446, CG5003, CG12225, CG7035, CG7139, CG17904, CG5045, CG7471, CG3061, CG6227, CG8000, CG6247, CG9910, CG4443, CG4578, CG1548, CG2747, CG17767, CG4026, CG5739, CG12000, CG10647, CG5604, CG10799, CG3051, CG17920, CG11901, CG6618, CG6827, CG8055, CG3860, CG10764, CG5790, CG11018, CG6304, CG5253, CG5413, CG8006
10	CG4767, CG3151, CG4948, CG8756, CG8529, CG17121, CG3624, CG18597, CG17124, CG11177, CG14681, CG9503, CG15736, CG6415, CG6711, CG3960, CG1960, CG4029, CG8222, CG8776, CG3183, CG18369, CG12298, CG17252, CG5467, CG5441, CG12941, CG9366, CG11395, CG7651, CG1250, CG18251, CG13345, CG5958, CG9862, CG17870, CG4230, CG16857, CG6712, CG2239, CG10137, CG9433, CG12261, CG5857, CG6066, CG7758, CG15097, CG8176, CG3274, CG13322, CG10670, CG13263, CG14477, CG13348, CG7493, CG2019, CG11341, CG13928, CG1244, CG7018, CG5699, CG8913, CG5941, CG14030, CG7110, CG17119, CG9134, CG10572, CG6882, CG10377, CG3711, CG6114, CG3174, CG6863, CG11168, CG7624, CG10737, CG17919, CG10611, CG3605, CG14789, CG14818, CG14039, CG8991, CG6376, CG18452, CG7396, CG9866, CG11906, CG2967, CG3246, CG3799, CG3218, CG3883, CG10446, CG5390, CG8678, CG10483, CG17323, CG6320, CG18069, CG1688, CG6767, CG17246, CG8730, CG12339, CG11023, CG1697, CG3090, CG14821, CG13515, CG9664, CG2640, CG5270, CG10426, CG8723, CG4341, CG12785, CG18227, CG11871, CG18210, CG12143, CG4096, CG12028, CG1922, CG9218, CG9676, CG5600, CG14653, CG8285, CG11201, CG10599, CG7910

Table, S1.b, Twelve, clusters, of, the, full, time, series

Class	Members
1	CG2079, CG7850, CG18597, CG6325, CG5914, CG7105, CG4962, CG7107, CG17332, CG1104, CG16820, CG6238, CG6981, CG12105, CG8756, CG7738, CG13868, CG10657, CG4302, CG12734, CG15811, CG18285, CG4229, CG6908, CG10433, CG10107, CG8846, CG5839, CG4370, CG1090, CG5326, CG11395, CG9336, CG16761, CG9338, CG13480, CG3344, CG17870, CG15560, CG2264, CG7456, CG3106, CG14989, CG3006, CG2145, CG5939, CG8568, CG6676, CG2505, CG7229, CG1106, CG3413, CG6836, CG3364, CG4791, CG9509, CG8864, CG9098, CG5772, CG9780, CG9510, CG9536, CG9665, CG4427, CG3829, CG7529, CG18512, CG15097, CG2736, CG9503, CG16858, CG1545, CG18076, CG6643, CG9701, CG15084, CG7074, CG8380, CG1623, CG11181, CG14994, CG3041, CG11605, CG7347, CG9201, CG1105, CG2239, CG4462, CG9512, CG15900, CG5875, CG9379, CG2143, CG10965, CG18409, CG10311, CG6794, CG2062, CG3879, CG15444, CG1114, CG17027, CG10562, CG14996, CG4556, CG5411, CG10830, CG11347, CG9012, CG2534, CG9619, CG2155, CG6667, CG9485, CG1208, CG14830, CG6023, CG12840, CG7651, CG18638, CG3262, CG7758, CG4643, CG9505, CG12089, CG10121, CG17156, CG10596, CG8975, CG8399, CG8547, CG9572, CG14528, CG3991, CG14207, CG3429, CG7365, CG18319, CG9155, CG11317, CG2669, CG4475, CG18358, CG6625, CG11221, CG4168, CG6465, CG1780, CG6740, CG7413, CG5140, CG5308, CG4645, CG10372, CG9821, CG6844, CG11477, CG8128, CG6292, CG3246, CG8909, CG5594, CG5783, CG18138, CG10346, CG9335, CG6702, CG12403, CG5797, CG6394, CG7576, CG8826, CG10706, CG5461, CG6236, CG6593, CG11403, CG6339, CG11883, CG10939, CG9071, CG14804, CG14024, CG17299, CG9646, CG11426, CG18227, CG4782, CG8174, CG1522, CG16928, CG1401, CG18452, CG6833, CG8084, CG3271, CG7392, CG11019, CG13830, CG8250, CG9195, CG11910, CG18061, CG1688, CG7708, CG2493, CG7143, CG4879, CG17919, CG13398, CG8485, CG10946, CG8109, CG7379, CG1605, CG9383, CG8171, CG4316, CG10137, CG7536, CG8912, CG6214, CG8907, CG2310, CG9031, CG11208, CG6678, CG8651, CG1848, CG12191, CG9947, CG12473, CG5469, CG10988, CG8247, CG6006, CG13777, CG3762, CG13773, CG3522, CG6941, CG11329, CG6453, CG3020, CG9633, CG10142, CG9164, CG17161, CG10428, CG1453, CG5202, CG4502, CG4232, CG1044, CG7238, CG12104, CG5776, CG7745, CG11715, CG5355, CG3324, CG18551, CG11791, CG10737, CG11960, CG3812, CG18533, CG14999, CG5067, CG17138, CG17064, CG8372, CG6868, CG11194, CG7149, CG13089, CG8347, CG3461, CG17369, CG14818, CG1763, CG4993, CG6723, CG12237, CG10255, CG3999, CG2144, CG14459, CG12598, CG10204, CG7101, CG9437, CG7066, CG10520, CG1109, CG6814, CG1815, CG5701, CG1416, CG7614, CG17994, CG3390, CG10385, CG1751, CG8931, CG5553, CG5643, CG6758, CG10209, CG4656, CG2176, CG5291, CG11148, CG3959, CG11191, CG8919, CG10961, CG9054, CG7671, CG17397, CG7264, CG9007, CG8261, CG3161, CG1071, CG17556, CG4257, CG9104, CG14821, CG2926, CG7359, CG9986, CG1474, CG6192, CG4909, CG17746, CG13605, CG10144, CG7948, CG8374, CG17509, CG2698, CG14211, CG7015, CG12942, CG7011, CG13718, CG3533, CG4617, CG7396, CG13345, CG8783, CG13322, CG12701, CG8444, CG5145, CG6905, CG17248, CG12082, CG1659, CG14224, CG6407, CG10149, CG11586,

	CG8156, CG2794, CG8825, CG2221, CG8287, CG16757, CG6147, CG11207, CG11625, CG2225, CG14442, CG12295, CG14969, CG2917, CG10609, CG3338, CG1388, CG5135, CG18112, CG17520, CG7940, CG11848, CG7623, CG18093, CG12238, CG11367, CG15435, CG18069, CG7846, CG12348, CG11330, CG10955, CG6406, CG17806, CG2934, CG9791, CG1954, CG5282, CG6674, CG3329, CG3223, CG2621, CG3004, CG4164, CG12878, CG4626, CG11871, CG3445, CG5877, CG10415, CG6098, CG5684, CG6718, CG10632, CG5884, CG2670, CG3291, CG4764, CG3138, CG10384, CG2019, CG3658, CG15312, CG3570, CG1088, CG8765, CG3060, CG3455, CG12127, CG9874, CG6939, CG10387, CG18214, CG12121, CG9356, CG5014, CG10795, CG2131, CG3309, CG3779, CG6754, CG15104, CG16868, CG5788, CG18324, CG3421, CG12316, CG2982, CG11274, CG17762, CG7007, CG8185, CG6773, CG7773, CG6742, CG5850, CG4586, CG18210, CG5284
2	CG17012, CG4033, CG8475, CG4266, CG4013, CG8806, CG4663, CG7445, CG15848, CG1124, CG10530, CG9603, CG11650, CG4818, CG4905, CG1733, CG8403, CG13787, CG18179, CG3558, CG6564, CG11142, CG11591, CG6821, CG14802, CG5358, CG13049, CG4221, CG12125, CG10475, CG8182, CG9411, CG5562, CG12763, CG4407, CG6296, CG12505, CG4649, CG4859, CG2663, CG5205, CG17682, CG11912, CG7953, CG18134, CG7924, CG16887, CG5584, CG5886, CG3756, CG7088, CG8358, CG18102, CG5973, CG5370, CG7090, CG4386, CG12789, CG1435, CG9331, CG1773, CG5325, CG1438, CG9466, CG4500, CG3648, CG5629, CG10131, CG6415, CG10912, CG6458, CG6467, CG8501, CG8272, CG14682, CG13841, CG4466, CG4354, CG4822, CG6169, CG18337, CG5413, CG11120, CG12703, CG1252, CG10128, CG6433, CG15288, CG3241, CG4122, CG8785, CG2070, CG12115, CG10374, CG8950, CG2040, CG10691, CG3066, CG10799, CG8242, CG4224, CG15390, CG13263, CG13627, CG10146, CG6664, CG4456, CG3625, CG7266, CG4524, CG4268, CG6170, CG18525, CG12011, CG1479, CG17754, CG6166, CG15658, CG10591, CG16701, CG2858, CG5428, CG2204, CG12045, CG15618, CG4233, CG14438, CG5770, CG16791, CG2081, CG11353, CG11509, CG2723, CG18140, CG5837, CG10814, CG14954, CG10630, CG6968, CG6173, CG1326, CG9761, CG4471, CG13184, CG8532, CG10672, CG9027, CG8479, CG3126, CG8605, CG1349, CG1548, CG7902, CG8376, CG15131, CG10619, CG8189, CG18019, CG17608, CG11967, CG3375, CG6639, CG7342, CG4760, CG10249, CG1112, CG7069, CG9717, CG6716, CG18550, CG11448, CG10416, CG18466, CG2835, CG3009, CG10595, CG18249, CG14766, CG3870, CG10077, CG11999, CG13340, CG6988, CG7433, CG10366, CG5407, CG1607, CG6936, CG8445, CG17203, CG2467, CG1309, CG12214, CG10536, CG4336, CG4592, CG17060, CG10873, CG17111, CG15512, CG5058, CG8067, CG11089, CG11309, CG9344, CG12918, CG10123, CG4746, CG2555, CG12088, CG7178, CG1429, CG10757, CG9019, CG4120, CG11470, CG7549, CG5793, CG3603, CG4700, CG8420, CG12819, CG13850, CG1583, CG9576, CG12253, CG15309, CG1539, CG8861, CG9021, CG18111, CG10218, CG10082, CG1374, CG11407, CG18022, CG12167, CG2056, CG5290, CG3989, CG6687, CG14548, CG8669, CG11790, CG3493, CG11010, CG3182, CG14433, CG1236, CG11299, CG17292, CG13551, CG13728, CG11712, CG5707, CG12896, CG1383, CG8503, CG6009, CG11567, CG5320, CG10671, CG14214, CG11880, CG1921, CG1216, CG7958, CG3054, CG8054, CG5677, CG10390, CG13907, CG1597, CG12284, CG11255, CG13782, CG13521, CG5575, CG6831, CG11992, CG17148, CG3327, CG1028, CG6841, CG3376, CG4589, CG10928, CG8144, CG7748, CG6094, CG10967, CG3356, CG7956, CG9796, CG17771, CG4180, CG17597, CG3108, CG7437, CG14889, CG14981, CG13221, CG7806, CG7624, CG1927, CG7122, CG11033, CG3139, CG5055, CG2060, CG15151, CG10130, CG1102, CG7183, CG8355, CG2681, CG1007, CG1444, CG10509, CG1532, CG3365, CG5871, CG10487, CG4581, CG8946, CG15340, CG2578, CG1358, CG17667, CG7821, CG8494, CG11267, CG6323, CG10072, CG7293, CG7125, CG7690, CG10039, CG6367, CG5201, CG14997, CG8486, CG6645, CG8118, CG3875, CG8057, CG11971, CG12744, CG10922, CG4823, CG5931, CG12251, CG12529, CG3564, CG10211, CG17602, CG10732, CG9798, CG14732, CG7123, CG6264, CG10593, CG17342, CG4677, CG14689, CG7865, CG4525, CG3793, CG7240, CG1225, CG17223, CG9812, CG7649, CG4341, CG10194, CG12273, CG13319, CG13784, CG6215, CG11388, CG13889, CG17841, CG1553, CG18398, CG18282, CG8348, CG1469, CG6714, CG1089, CG1977, CG9763, CG6829, CG6647, CG11685, CG11297, CG12002, CG17840, CG4875, CG3753, CG4678, CG7159, CG5966, CG9707, CG1408, CG16745, CG2862, CG1651, CG6827, CG12101, CG11595, CG14899, CG4067, CG1739, CG4275, CG5695, CG8616, CG1849, CG11428, CG11888, CG1856, CG5345, CG3715, CG8775, CG10373, CG17082, CG1449, CG18253, CG12156, CG9766, CG2867, CG13458, CG2102, CG7509, CG6050, CG5953, CG10439, CG7291, CG3268, CG2139, CG8884, CG7448, CG10462, CG13917, CG6272, CG6342, CG1635, CG11206, CG2641, CG7518, CG6193, CG6044, CG5084, CG3666, CG4114, CG8199, CG1514, CG4447, CG11454, CG12204, CG2151, CG5163, CG1782, CG1794, CG12075, CG11658, CG1472, CG9186, CG2694, CG9933, CG15228, CG11526, CG9484, CG10325, CG13185, CG12261, CG15667, CG7525, CG15100, CG16941, CG6673, CG4619, CG9268, CG13598, CG4262, CG7296, CG4036, CG8782, CG7093, CG10585, CG2522, CG17348, CG2507, CG8470, CG3304, CG8862, CG8384, CG8073, CG1903, CG14771, CG7642, CG17524, CG8237, CG10293, CG7834, CG3257, CG7048, CG6695, CG17035, CG9216, CG4768, CG14938, CG7148, CG3973, CG7800, CG4538, CG4389, CG11427, CG4261, CG4710, CG8215, CG14723, CG10881, CG6725, CG12894, CG7469, CG13130, CG5047, CG14800, CG3090, CG7438, CG6028, CG18497, CG6145, CG15609, CG5044, CG8026, CG9453, CG9577, CG6450, CG10289, CG5805, CG4848, CG3770, CG1818, CG7656, CG5248, CG13603, CG7163, CG3270, CG11807, CG6699, CG7586, CG13647, CG6016, CG4703, CG7224, CG7613, CG3971, CG3988, CG3059, CG1888, CG6815, CG14782, CG3978, CG4012, CG9985, CG9026, CG1239, CG7654, CG8552, CG13890, CG18677, CG3441, CG3810, CG5186, CG9573, CG2304, CG4761, CG14992, CG8139, CG15426, CG7203, CG5486, CG16705, CG4199, CG6369, CG11763, CG11276, CG7029, CG3045, CG3764, CG8819, CG7795, CG5721, CG9746, CG10977, CG8046, CG10916, CG7041, CG1964, CG17025, CG4608, CG12876, CG1963, CG10577, CG8230, CG10701, CG5989, CG4008, CG8818, CG6302, CG11035, CG7391, CG7887, CG8031, CG6838, CG7062, CG11169, CG15013, CG6313, CG5846, CG17485, CG7484, CG17508, CG11045, CG3920, CG1814, CG4963, CG6224, CG5037, CG8575, CG9047, CG1467, CG8954, CG17765, CG6811, CG9497, CG4751, CG6984, CG12582, CG6355, CG10704, CG15217, CG8159, CG11537, CG4951, CG9056, CG16778, CG6388, CG10764, CG14045, CG7177, CG12090, CG3178, CG11534, CG8460, CG8055, CG9958, CG9660, CG7099, CG2006, CG3312, CG4974, CG16932, CG2177, CG13812, CG8815, CG11964, CG4925, CG9337, CG1322, CG5275, CG8583, CG9415, CG8601, CG4842, CG3811, CG9318, CG5670, CG9559, CG12305, CG10408, CG18546,

	CG3868, CG17090, CG4668, CG4644, CG7814, CG8059, CG7100, CG2226, CG11210, CG9005, CG12016, CG4124, CG10210, CG5127, CG1086, CG6528, CG17046, CG10846, CG12189, CG8980, CG9804, CG9709, CG12096, CG12263, CG14296, CG1893, CG11887, CG7511, CG7955, CG6911, CG8722, CG2358, CG5841, CG7961, CG14813, CG1846, CG4317, CG7507, CG6923, CG12753, CG17611, CG7319, CG6116, CG15012, CG6204, CG17686, CG10295, CG11462, CG13667, CG18582, CG4061, CG3662, CG6510, CG2716, CG3160, CG9543, CG1371, CG12099, CG5742, CG1799, CG11170, CG18011, CG2049, CG4347, CG1017, CG6995, CG1937, CG5862, CG5815, CG3411, CG12004, CG1233, CG5706, CG5166, CG7927, CG1770, CG11334, CG4062, CG5382, CG11290, CG14991, CG15386, CG6922, CG12283, CG3738, CG15772, CG8205, CG8110, CG3409, CG8243, CG4088, CG9852, CG6345, CG3874, CG17920, CG1898, CG9140, CG9934, CG15081, CG5412, CG8643, CG13503, CG8974, CG17596, CG11869, CG15814, CG15099, CG3902, CG11700, CG7962, CG1730, CG10021, CG3403, CG2086, CG1952, CG10199, CG5404, CG9288, CG5961, CG1810, CG4126, CG13483, CG7383, CG9799, CG17894, CG1806, CG3723, CG12168, CG14721, CG9099, CG9916, CG11958, CG5196, CG11980, CG3590, CG3173, CG1099, CG16844, CG15440, CG2095, CG7987, CG5080, CG6668, CG1049, CG1055, CG15433, CG3460, CG10537, CG2708, CG4629, CG5287, CG6767, CG4186, CG4078, CG9305, CG3992, CG3143, CG10755, CG9884, CG3423, CG15118, CG12532, CG18352, CG4276, CG9139, CG3499, CG11770, CG11183, CG12832, CG7142, CG18176, CG9779, CG3857, CG5206, CG14231, CG2028, CG8431, CG5348, CG9398, CG11482, CG1582, CG10115, CG5651, CG4763, CG4966, CG8814, CG2179, CG9325, CG7499, CG7925, CG5714, CG11806, CG3213, CG15437, CG1824, CG10605, CG8789, CG1534, CG5191, CG3663, CG8416, CG5417, CG5073, CG10647, CG9208, CG3093, CG5482, CG6603, CG8201, CG12163, CG14026, CG4557, CG7414, CG4713, CG4729, CG3682, CG11316, CG1559, CG9317, CG7097, CG11414, CG14195, CG1454, CG12076, CG4211, CG8657, CG7364, CG11098, CG9528, CG7985, CG5373, CG5859, CG15557, CG11919, CG8092, CG18858, CG5036, CG8565, CG12007, CG2448, CG5108, CG6543, CG3530, CG3615, CG9144, CG6851, CG6615, CG13630, CG10860, CG12781, CG17514, CG8286, CG9063, CG18616, CG17712, CG18459, CG6665, CG1241, CG6606, CG5576, CG3731, CG4472, CG7734, CG3335, CG18143, CG8969, CG7967, CG6156, CG4182, CG9775, CG17077, CG10711, CG18627, CG18476, CG7358, CG7672, CG5053, CG8777, CG10443, CG6775, CG18462, CG6159, CG12071, CG8453, CG7057, CG5215, CG14461, CG12212, CG1201, CG3668, CG1107, CG4542, CG5251, CG1200, CG6618, CG1707, CG16944, CG11622, CG6706, CG7324, CG5004
3	CG9176, CG8135, CG3504, CG11473, CG1263, CG18404, CG5119, CG12178, CG10810, CG17617, CG4847, CG4679, CG10570, CG17210, CG3306, CG8329, CG6186, CG9699, CG5095, CG1462, CG10589, CG4799, CG10126, CG10097, CG13865, CG10953, CG3772, CG3330, CG16772, CG4123, CG3368, CG7557, CG2803, CG8994, CG7077, CG7096, CG4265, CG4715, CG9377, CG2750, CG9734, CG6306, CG9581, CG2096, CG1108, CG3966, CG12373, CG6730, CG2077, CG10553, CG12120, CG4837, CG8576, CG5807, CG2045, CG4392, CG1787, CG3121, CG4961, CG17238, CG6652, CG1535, CG18278, CG3982, CG2604, CG8918, CG11400, CG5534, CG6045, CG1827, CG2150, CG16989, CG3127, CG6980, CG10192, CG16959, CG9828, CG5107, CG16783, CG8723, CG7655, CG1655, CG9392, CG10599, CG1742, CG8572, CG12321, CG9741, CG4390, CG10446, CG6614, CG5105, CG9898, CG2528, CG3924, CG1791, CG14209, CG5164, CG1670, CG3287, CG8509, CG5826, CG9009, CG10283, CG7563, CG5045, CG9896, CG8866, CG13124, CG11955, CG6265, CG18539, CG9708, CG3961, CG8285, CG6233, CG15445, CG7590, CG17759, CG13714, CG12220, CG11146, CG1506, CG2088, CG8725, CG5112, CG7607, CG3906, CG6575, CG10622, CG9067, CG9878, CG10186, CG11738, CG9772, CG14080, CG9808, CG17452, CG3881, CG10238, CG11446, CG2657, CG3195, CG4742, CG7263, CG4446, CG10254, CG3350, CG14472, CG3644, CG3743, CG7515, CG2952, CG3702, CG7939, CG12162, CG2061, CG7277, CG5448, CG12123, CG12428, CG4087, CG5686, CG8155, CG9715, CG8609, CG1116, CG10859, CG7479, CG10997, CG9282, CG3269, CG8759, CG3353, CG15608, CG6518, CG1081, CG8715, CG14617, CG6912, CG5013, CG6285, CG8056, CG17352, CG18316, CG10539, CG10497, CG7483, CG2852, CG18238, CG10409, CG8944, CG17952, CG1883, CG3751, CG1973, CG5741, CG4897, CG3314, CG18623, CG17420, CG1059, CG8409, CG9469, CG18662, CG6197, CG8332, CG1210, CG10777, CG10484, CG1115, CG4578, CG12775, CG3922, CG8531, CG6903, CG2246, CG5429, CG7977, CG13298, CG7945, CG7899, CG9075, CG8857, CG12232, CG6779, CG3618, CG6523, CG12727, CG4071, CG4759, CG5502, CG17521, CG6141, CG15442, CG10944, CG7701, CG7702, CG12210, CG3825, CG5821, CG8495, CG15086, CG6283, CG10494, CG12106, CG9119, CG10281, CG9091, CG7490, CG14630, CG18174, CG2190, CG2998, CG14792, CG7283, CG11282, CG6686, CG5920, CG12740, CG10652, CG12139, CG12638, CG8370, CG8615, CG11248, CG17184, CG11352, CG7129
4	CG3612, CG15520, CG2287, CG5596, CG17927, CG4795, CG9432, CG3116, CG16707, CG7727, CG11916, CG4468, CG9441, CG5904, CG7443, CG7028, CG10949, CG7565, CG9090, CG8154, CG13240, CG18251, CG17903, CG10888, CG8256, CG9244, CG11303, CG10245, CG6305, CG18020, CG1744, CG10226, CG1103, CG5889, CG1163, CG4899, CG1721, CG12207, CG3151, CG16882, CG2171, CG9480, CG9431, CG17272, CG6320, CG11963, CG12054, CG8732, CG9400, CG6043, CG4769, CG10550, CG5262, CG6123, CG1889, CG13356, CG11236, CG6022, CG6126, CG5903, CG4461, CG18255, CG1537, CG17800, CG1826, CG14290, CG7321, CG4353, CG4945, CG8024, CG14724, CG2140, CG10693, CG13387, CG5449, CG9306, CG3446, CG4144, CG1065, CG4972, CG13796, CG6105, CG12606, CG5270, CG4692, CG14940, CG9865, CG6042, CG3560, CG6439, CG12955, CG5261, CG11455, CG4975, CG8320, CG8740, CG9264, CG9438, CG10233, CG5125, CG6343, CG11354, CG1970, CG12079, CG6622, CG17246, CG4094, CG8511, CG17926, CG9762, CG4307, CG10483, CG5532, CG12233, CG13323, CG1915, CG7580, CG13219, CG4894, CG5071, CG9131, CG1715, CG9921, CG2457, CG11661, CG4001, CG10675, CG5172, CG11914, CG15387, CG6550, CG9172, CG5523, CG6020, CG7757, CG14490, CG14653, CG6455, CG7430, CG1922, CG10664, CG7897, CG5703
5	CG3245, CG5571, CG9136, CG5790, CG10202, CG2127, CG11475, CG14740, CG6304, CG6456, CG12699, CG4714, CG8040, CG8701, CG12136, CG3492, CG4750, CG1340, CG9920, CG11018, CG4836, CG9133, CG17567, CG3380, CG1540, CG14735, CG1324, CG5538, CG14355, CG5089, CG9314, CG5048, CG8462,

	CG1394, CG1979, CG2830, CG7815, CG17349, CG8136, CG8043, CG10752, CG14183, CG6372, CG7886, CG3315, CG6541, CG17470, CG6441, CG17838, CG5398, CG17302, CG10934, CG1999, CG8006, CG14786, CG6790, CG11703, CG15208, CG9803, CG9323, CG18418, CG2267, CG6332, CG10742, CG10138, CG18568, CG17450, CG7669, CG9483, CG9130, CG17645, CG5735, CG8683, CG6255, CG6761, CG10631, CG7387, CG1314, CG6130, CG7634, CG14021, CG10999, CG6059, CG8023, CG1193, CG7045, CG5539, CG5443, CG7742, CG13030, CG7366, CG14891, CG4983, CG12860, CG5983, CG3253, CG1835, CG17769, CG3565, CG10841, CG9218, CG2616, CG7794, CG7131, CG12028, CG4995, CG5755, CG8838, CG3523, CG4681, CG2385, CG11656, CG2854, CG15360, CG2921, CG4810, CG2209, CG3092, CG8772, CG3557, CG13280, CG17010, CG8817, CG9531, CG11908, CG10561, CG9284, CG1615, CG7816, CG3809, CG2149, CG1760, CG18396, CG5237, CG11074, CG9712, CG6661, CG3124, CG7415, CG12605, CG16716, CG4767, CG12907, CG15867, CG2907, CG8362, CG17564, CG8508, CG18218, CG3085, CG8619, CG1495, CG10365, CG9861, CG15891, CG11404, CG7441, CG4669, CG6180, CG8278, CG4913, CG13330, CG10854, CG11023, CG6670, CG12423, CG9010, CG10171, CG14718, CG5835, CG8257, CG3544, CG11201, CG7722, CG1274, CG4955, CG16979, CG9313, CG11546, CG4323, CG16972, CG1009, CG8525, CG5450, CG1146, CG13918, CG2046, CG3074, CG6230, CG3483, CG9580, CG12118, CG9151, CG6470, CG6405, CG8813, CG8979, CG8981, CG14995, CG8529, CG5555, CG1317, CG18000, CG7783, CG13604, CG3033, CG12184, CG4420, CG3748, CG7251, CG9085, CG10750, CG12770, CG9554, CG17492, CG7999, CG4059, CG7841, CG8739, CG15892, CG16833
6	CG4564, CG12235, CG2107, CG12211, CG5692, CG4929, CG6544, CG8122, CG8736, CG1803, CG9686, CG4784, CG7776, CG17052, CG8483, CG3616, CG12268, CG3331, CG7983, CG17549, CG17843, CG2016, CG8888, CG14235, CG7874, CG11390, CG10241, CG2082, CG9538, CG4107, CG9591, CG12449, CG10026, CG9450, CG10159, CG3541, CG7399, CG12846, CG11459, CG4250, CG4757, CG9456, CG3864, CG2198, CG1468, CG5173, CG9535, CG4375, CG3847, CG15855, CG13848, CG5390, CG2233, CG10816, CG2789, CG1600, CG6563, CG8913, CG17081, CG9363, CG9134, CG7230, CG8766, CG1516, CG17121, CG3097, CG2671, CG4321, CG4889, CG3566, CG4384, CG9364, CG7895, CG1378, CG6770, CG7434, CG11456, CG12612, CG7470, CG9676, CG3950, CG3571, CG5397, CG6819, CG11970, CG3129, CG5168, CG1521, CG12050, CG8730, CG6234, CG7635, CG12800, CG7571, CG1121, CG1101, CG6057, CG4526, CG12040, CG5600, CG10806, CG11100, CG5758, CG10228, CG3318, CG12240, CG6424, CG4376, CG6863, CG3936, CG17137, CG3192, CG17228, CG4891, CG15613, CG5888, CG7400, CG8855, CG9769, CG5405, CG4082, CG12143, CG7220, CG8633, CG9839, CG9312, CG1078, CG8896, CG17506, CG6227, CG8854, CG8606, CG14178, CG6711, CG11280, CG7720, CG11823, CG9507, CG7185, CG3726, CG2916, CG4039, CG7600, CG1523, CG4947, CG11860, CG15427, CG1133, CG4722, CG1754, CG17836, CG15101, CG3359, CG6620, CG10206, CG8995, CG7538, CG9885, CG14681, CG10800, CG3427, CG5930, CG5175, CG1363, CG6987, CG10347, CG5393, CG10327, CG5363, CG8060, CG1637, CG1658, CG4026, CG10230, CG4206, CG5009, CG2674, CG18350, CG8049, CG4455, CG10080, CG9135, CG9198, CG3238, CG10286, CG12306, CG13384, CG2328, CG3415, CG4826, CG2530, CG7524, CG6187, CG12372, CG17676, CG1911, CG10837, CG3333, CG11451, CG1070, CG16936, CG12006, CG12369, CG10108, CG12236, CG4432, CG1395, CG5522, CG5455, CG12389, CG2956, CG4029, CG12702, CG8920, CG17544, CG5848, CG6247, CG7528, CG5258, CG15736, CG13096, CG11266, CG3303, CG4096, CG17736, CG4424, CG11212, CG1943, CG1578, CG11177, CG3221, CG12138, CG5809, CG7788, CG4192, CG10391, CG3689, CG13849, CG1227, CG17367, CG5383, CG10341, CG4510, CG12339, CG4857, CG1676, CG4454, CG8793, CG3962, CG4817, CG3696, CG4108, CG10364, CG5198, CG9723, CG8929, CG14222, CG4898, CG1228, CG1041, CG18178, CG1074, CG5995, CG2968, CG2199, CG7281, CG16805, CG5353, CG8079, CG9300, CG7664, CG6701, CG5857, CG5784, CG1381, CG2843, CG5126, CG12301, CG14641, CG3619, CG10441, CG9663, CG17689, CG11228, CG2950, CG17273, CG12052, CG12022, CG9188, CG12179, CG11387, CG9018, CG7421, CG14895, CG8114, CG10627, CG5694, CG1710, CG17170, CG10990, CG8833, CG10355, CG6226, CG5190, CG8621, CG12130, CG8928, CG8013, CG1901, CG9776, CG17947, CG11837, CG7831, CG3851, CG4274, CG8553, CG18362, CG5591, CG7838, CG14657, CG5965, CG17252, CG10545, CG8625, CG13425, CG8611, CG1528, CG7004, CG3428, CG2050, CG1866, CG1591, CG6724, CG9630, CG7878, CG17958, CG3820, CG5919, CG4831, CG2984, CG10016, CG9107, CG4201, CG6066, CG14941, CG6994, CG11877, CG6582, CG6962, CG17912, CG6331, CG9304, CG4263, CG5676, CG10267, CG6884, CG1240, CG6712, CG9177, CG4527, CG11935, CG5444, CG6391, CG8367, CG6501, CG8149, CG6316, CG6359, CG15439, CG10739, CG7845, CG12749, CG7055, CG5589, CG6538, CG6850, CG1753, CG7872, CG2899, CG11063, CG9028, CG3645, CG17493, CG5941, CG4747, CG11525, CG10689, CG3594, CG8892, CG5739, CG8289, CG10161, CG10212, CG2905, CG9754, CG7081, CG8233, CG11897, CG9166, CG2691, CG4260, CG11866, CG17938, CG5640, CG10528, CG15694, CG2244, CG7581, CG10043, CG11417, CG12941, CG8441, CG10590, CG3744, CG7986, CG4528, CG10712, CG2446, CG6556, CG11990, CG18622, CG4571, CG8705, CG7993, CG7102, CG4602, CG2017, CG7764, CG8108, CG10978, CG12135, CG11988, CG9862, CG6896, CG3480, CG9768, CG17454, CG1420, CG15532, CG7892, CG12759, CG10203, CG16901, CG3735, CG12202, CG5899, CG8309, CG4200, CG2890, CG8151, CG14939, CG7926, CG8326, CG13201, CG12134, CG7682, CG6418, CG11130, CG13626, CG12124, CG9967, CG16812, CG7564, CG8631, CG14216, CG10139, CG5808, CG10417, CG3018, CG6860, CG7275, CG10702, CG1587, CG3274, CG3941, CG4141, CG7110, CG9984, CG10065, CG4069, CG1913, CG6210, CG8617, CG1244, CG16753, CG10572, CG11486, CG2031, CG10851, CG2097, CG2091, CG17358, CG1648, CG7946, CG10754, CG1142, CG10565, CG8153, CG7338, CG8295, CG14709, CG5456, CG7494, CG8280, CG4659, CG18124, CG6502, CG8378, CG7351, CG2831, CG7839, CG10376, CG7704, CG2922, CG4057, CG5018, CG10009, CG7824, CG18273, CG7035, CG4699, CG6677, CG17484, CG7558, CG4258, CG5787, CG1671, CG18177, CG7269, CG9373, CG7859, CG6963, CG8983, CG4654, CG4236, CG5510, CG11820, CG3710, CG15010, CG1906, CG5954, CG9866, CG2168, CG13097, CG10923, CG5649, CG1542, CG10274, CG9805, CG5786, CG6584, CG10473, CG8882, CG4364, CG7843, CG3704, CG2682, CG9277, CG3613, CG7262, CG6474, CG2945, CG3918, CG4621, CG17436, CG7725, CG12750, CG9556, CG12000, CG12085, CG9680, CG7752, CG7334, CG1925, CG2182, CG3605, CG11154, CG13929, CG6395, CG4813, CG14650, CG2503, CG8000, CG12234, CG4878, CG5193, CG6854, CG18426, CG4152, CG4943, CG12230, CG9696, CG6375, CG14226, CG7098, CG10392,

	CG4806, CG4852, CG11104, CG7626, CG6015, CG4622, CG2260, CG4118, CG8877, CG4985, CG18592, CG3180, CG18683, CG7546, CG17153, CG12340, CG7288, CG4563, CG2976, CG18608, CG4184, CG12785, CG6241, CG10686, CG5003, CG5422, CG5099, CG12113, CG9797, CG4217, CG9666, CG2034, CG11901, CG11375, CG4453, CG3218, CG6506, CG12152, CG3203, CG3073, CG6254, CG9238, CG2469, CG8902, CG11723, CG1677, CG2925, CG1646, CG5519, CG9910, CG17033, CG3634, CG7583, CG8571, CG11678, CG14722, CG4548, CG16788, CG1500, CG1765, CG7728, CG8749, CG4303, CG12225, CG7705, CG2712, CG7162, CG1596, CG7109, CG7139, CG8580, CG7471
7	CG8279, CG2055, CG12290, CG5942, CG17998, CG15509, CG10155, CG9704, CG17977, CG6483, CG10472, CG12374, CG17814, CG11466, CG13095, CG10514, CG6416, CG10513, CG2677, CG7216, CG7065, CG8560, CG1865, CG18444, CG9682, CG4533, CG9672, CG12279, CG14032, CG6484, CG7002, CG14318, CG17534, CG3168, CG3481, CG14904, CG4612, CG10143, CG18369, CG16727, CG5955, CG7910, CG7050, CG4377, CG4719, CG1412, CG5150, CG18033, CG17875, CG6640, CG5813, CG18317, CG15068, CG12182, CG8345, CG7070, CG10492, CG1536, CG18522, CG8588, CG2196, CG9649, CG16719, CG3835, CG6440, CG3301, CG5165, CG8251, CG10842, CG3372, CG7021, CG9308, CG7920, CG10042, CG8012, CG10116, CG5867, CG7231, CG1168, CG8577, CG3790, CG6376, CG7913, CG10426, CG1220, CG10527, CG11325, CG5945, CG10383, CG3690, CG4609, CG4355, CG11094, CG7493, CG9889, CG11841, CG9451, CG11425, CG8216, CG3752, CG14205, CG14704, CG8600, CG10833, CG8314, CG1862, CG2640, CG9813, CG1552, CG5288, CG6942, CG3285, CG5748, CG7611, CG9448, CG3529, CG6958, CG6398, CG3795, CG9467, CG8733, CG6503, CG17654, CG8591, CG6432, CG9836, CG1726, CG1839, CG4402, CG9445, CG7873, CG17437, CG7147, CG17723, CG11293, CG5740, CG6315, CG8620, CG16987, CG9932, CG12355, CG3724, CG5466, CG3722, CG7022, CG5154, CG17884, CG5506, CG6845, CG1021, CG8298, CG14125, CG5627, CG12375, CG13388, CG3091, CG8086, CG11344, CG11782, CG14228, CG13594, CG18314, CG18107, CG6014, CG1640, CG5687, CG8602, CG4653, CG5946, CG9086, CG16973, CG2163, CG18403, CG3727, CG9664, CG3937, CG3885, CG14896, CG10291, CG4416, CG6128, CG7829, CG5279, CG4491, CG1354, CG6058, CG12982, CG7068, CG10719, CG15688, CG8232, CG6282, CG3075, CG7049, CG5926, CG8654, CG17822, CG4685, CG1530, CG10217, CG8451, CG15279, CG10947, CG5588, CG4037, CG4694, CG12213, CG8212, CG8288, CG13800, CG5109, CG8195, CG7082, CG3200, CG7803, CG14233, CG5212, CG6335, CG13852, CG17975, CG6791, CG3878, CG5712, CG1057, CG11737, CG6509, CG15610, CG4288, CG4682, CG14208, CG11711, CG3845, CG3187, CG1905, CG1918, CG3998, CG9764, CG11232, CG9209, CG7158, CG4006, CG13620, CG16724, CG5010, CG10555, CG8613, CG8637, CG5634, CG17336, CG7058, CG2218, CG17809, CG10508, CG2765, CG7012, CG12466, CG10053, CG9391, CG9008, CG2135, CG2971, CG10966, CG10869, CG9403, CG1512, CG11968, CG15105, CG9886, CG3308, CG9162, CG3290, CG3883, CG9343, CG14008, CG3955, CG5505, CG1407, CG10538, CG6734, CG10084, CG1965, CG5902, CG18212, CG5868, CG7188, CG14213, CG12170, CG8032, CG3832, CG6446, CG6290, CG11798, CG10579, CG14646, CG4000, CG8179, CG5845, CG6771, CG9653, CG6382, CG11371, CG5530, CG4428, CG8593, CG9749, CG13421, CG5102, CG10279, CG9088, CG1830, CG15668, CG8401, CG11061, CG7766, CG16982, CG1212, CG14408, CG8312, CG2819, CG6707, CG3305, CG4766, CG11940, CG17034, CG2818, CG8549, CG8083, CG3086, CG3458, CG8465, CG11874, CG3584, CG9245, CG18494, CG17904
8	CG1161, CG2985, CG8318, CG11779, CG1342, CG6882, CG4702, CG7675, CG8684, CG3061, CG4664, CG5431, CG4720, CG7227, CG5779, CG2666, CG9689, CG1681, CG8596, CG1441, CG9443, CG9520, CG1153, CG3624, CG2718, CG7144, CG8222, CG5803, CG10898, CG8468, CG18065, CG3632, CG6611, CG6385, CG6959, CG3152, CG9795, CG2767, CG2961, CG10621, CG11642, CG10236, CG17124, CG4841, CG12467, CG2685, CG5123, CG2054, CG12758, CG10882, CG7724, CG2970, CG16857, CG17767, CG4311, CG5441, CG2902, CG12311, CG1969, CG3799, CG4829, CG1657, CG7540, CG9342, CG6977, CG11836, CG7896, CG1471, CG8428, CG8660, CG3322, CG6038, CG15165, CG7388, CG9148, CG7805, CG8306, CG3758, CG10849, CG3174, CG3652, CG10193, CG13928, CG12706, CG4572, CG2996, CG8678, CG10641, CG6572, CG8639, CG1697, CG10382, CG9042, CG6746, CG8676, CG9629, CG9489, CG14712, CG9165, CG15002, CG10060, CG18495, CG5840, CG10151, CG11945, CG12869, CG12035, CG8536, CG6199, CG3556, CG17921, CG7802, CG3399, CG11341, CG5406, CG10733, CG1554, CG17260, CG5822, CG8707, CG8363, CG11188, CG9972, CG9968, CG11200, CG12008, CG17370, CG11961, CG7486, CG10106, CG1451, CG2103, CG13515, CG8121, CG17632, CG2967, CG12030, CG7480, CG3608, CG6033, CG5394, CG4443, CG3184, CG4928, CG12091, CG7830, CG8389, CG8229, CG5170, CG13348, CG2485, CG6608, CG6114, CG4618, CG8190, CG6448, CG3528, CG14789, CG5915, CG4948, CG6822, CG17711, CG3156, CG17737, CG10277, CG3860, CG3400, CG2918, CG4426, CG10948, CG11247, CG17323, CG10076, CG5729, CG8196, CG3328, CG5802, CG7217, CG7199, CG7503, CG12131, CG8991, CG8368, CG6404, CG2216, CG11547, CG3877, CG4523, CG18380, CG7018, CG1902, CG7502, CG9319, CG11050, CG5094, CG5468, CG17888, CG2747, CG8422, CG4662, CG9159, CG5032, CG3967, CG3282, CG2994, CG3135, CG11982, CG8887, CG3459, CG7206, CG5231, CG15513, CG5604, CG10897, CG4230, CG3620, CG1844, CG8266, CG1303, CG3289, CG1287, CG7510, CG17255, CG6054, CG6899, CG3664, CG10985, CG4577, CG6735, CG8743, CG1737, CG14683, CG3051, CG10566, CG8045, CG1345, CG9423, CG7073, CG6495, CG1277, CG9553, CG3887, CG9908
9	CG6222, CG8379, CG5654, CG16926, CG8505, CG3191, CG9425, CG8632, CG7176, CG10237, CG4778, CG15634, CG10200, CG2330, CG1743, CG14455, CG16758, CG5853, CG7981, CG5656, CG7780, CG2727, CG9568, CG11064, CG3823, CG5958, CG10248, CG10512, CG11440, CG3960, CG3281, CG15818, CG15095, CG7737, CG3711, CG9366, CG17999, CG17646, CG8352, CG13432, CG3510, CG5467, CG9834, CG4827, CG15319, CG9506, CG5699, CG14808, CG17835, CG13770, CG5568, CG11660, CG8776, CG8960, CG5100, CG9362, CG15835, CG14477, CG18437, CG5452, CG4877, CG4482, CG9953, CG10444, CG11005, CG7425, CG3102, CG5940, CG4639, CG1250, CG8507, CG6425, CG7801, CG4357, CG12756, CG8290, CG5880, CG12117, CG8569, CG4968, CG1647, CG11906, CG9321, CG5490, CG10938, CG3938, CG15893, CG1519, CG14025, CG5726, CG1636, CG2186, CG5581, CG12892, CG4166, CG1091, CG7407, CG1490, CG2076,

	CG5632, CG17678, CG17018, CG14039, CG1745, CG17129, CG4916, CG3183, CG1024, CG8116, CG6157, CG1602, CG8357, CG17119, CG5026, CG11484, CG15737, CG6719, CG3910, CG11168, CG9705, CG9433, CG7979, CG5263, CG14965, CG11471, CG8435, CG7530, CG8433, CG5541, CG2943, CG13832, CG13840, CG1307, CG1624, CG6258, CG9858, CG10018, CG12081, CG9293, CG5924, CG8548, CG5590, CG6144, CG5203, CG12109, CG8448, CG7825, CG9239, CG12737, CG10038, CG1962, CG8961, CG5971, CG8068, CG5785, CG7832, CG5462, CG8390, CG12244, CG5580, CG2947, CG4488, CG14303, CG2864, CG6219, CG15804, CG10033, CG2051, CG4920, CG9925, CG8187, CG1503, CG2258, CG9241, CG14005, CG1884, CG1960, CG18004, CG17294, CG2678, CG1643, CG3654, CG1708, CG8856, CG11856, CG12298, CG1034, CG7212, CG8253, CG3876, CG3157, CG14749, CG3082, CG4495, CG3628, CG11490, CG3167, CG11560, CG5174, CG4032, CG2910, CG1404, CG2790, CG6549, CG9231, CG12276, CG7663, CG3995, CG3021, CG7730, CG11808, CG14764, CG12317, CG3273, CG7869, CG5608, CG3208, CG1800, CG1716, CG10670, CG4949, CG4281, CG12363, CG1275, CG7197, CG3561, CG4300, CG1832, CG10954, CG11397, CG10057, CG4973, CG3342, CG4497, CG6251, CG9126, CG3889, CG11504, CG3249, CG17322, CG3024, CG8284, CG4365, CG10377, CG4785, CG7597, CG11055, CG14030, CG14967, CG11242, CG10640, CG18145, CG6005, CG9062, CG11771, CG6363, CG7357, CG18042, CG18041, CG8924, CG2829, CG4867, CG7974, CG1515, CG1868, CG11596, CG1603, CG6542, CG17540, CG16892, CG11993, CG9213, CG1836, CG9890, CG12132, CG1341, CG5222, CG7853, CG7467, CG16798, CG6961, CG1487, CG3508, CG15112, CG8001, CG7146, CG6401, CG9949, CG8301, CG11943, CG4050, CG6311, CG6179, CG5146, CG7133, CG5639, CG10563, CG15817, CG4865, CG8176, CG9242, CG17681, CG1896, CG1218, CG5720, CG5991, CG9418, CG3497, CG17159, CG1976, CG2087, CG6422, CG8988, CG3511, CG8949, CG2158, CG9351, CG9446, CG1258
10	CG7314, CG7906, CG11186, CG4214, CG8175, CG7160, CG2707, CG12304, CG3397, CG8677, CG10658, CG6788, CG15009, CG13223, CG10370, CG6539, CG6745, CG4449, CG11275, CG6287, CG1746, CG6273, CG4999, CG9089, CG6206, CG4790, CG17752, CG11128, CG6662, CG4900, CG12177, CG14768, CG5002, CG14886, CG9057, CG4267, CG8947, CG1942, CG6319, CG7340, CG2048, CG7660, CG8603, CG7111, CG4600, CG18455, CG3297, CG5508, CG6656, CG8592, CG9523, CG8727, CG2025, CG7135, CG17469, CG5184, CG8735, CG6293, CG9924, CG3856, CG2903, CG18593, CG17515, CG5194, CG4604, CG11654, CG8231, CG7908, CG5938, CG17309, CG16742, CG10724, CG5780, CG6276, CG7995, CG10840, CG17603, CG11139, CG10308, CG1822, CG9578, CG8180, CG4535, CG8668, CG4919, CG3567, CG12024, CG2331, CG11278, CG7777, CG10222, CG7717, CG13607, CG14971, CG4422, CG2913, CG8714, CG1691, CG8062, CG13343, CG17274, CG6967, CG11171, CG6757, CG6692, CG11259, CG3042, CG3036, CG8240, CG5081, CG3172, CG5675, CG16817, CG6672, CG2162, CG4872, CG4084, CG11783, CG13671, CG2478, CG1464, CG17259, CG14444, CG4979, CG10716, CG6203, CG11804, CG10731, CG5844, CG17149, CG10234, CG2856, CG17818, CG7765, CG10420, CG10602, CG2675, CG9537, CG1489, CG10825, CG7627, CG17565, CG8235, CG9670, CG4735, CG11844, CG6605, CG4035, CG18543, CG7108, CG7103, CG4196, CG4030, CG2147, CG10797, CG2699, CG5977, CG12795, CG13397, CG4162, CG12397, CG13853, CG7033, CG7417, CG5916, CG13316, CG7531, CG7168, CG5495, CG7628, CG14100, CG15486, CG5374, CG8351, CG1092, CG4585, CG12015, CG3800, CG17019, CG7935, CG1938, CG3279, CG1100, CG13601, CG9246, CG1951, CG8439, CG4338, CG8440, CG6087, CG5378, CG5289, CG8402, CG3416
11	CG9345, CG6772, CG18490, CG6933, CG3672, CG15093, CG8342, CG9759, CG1809, CG5001, CG7252, CG13279, CG3588, CG6496, CG5567, CG8774, CG1774, CG10154, CG5887, CG6906, CG3987, CG4429, CG5597, CG7567, CG9675, CG10863, CG3332, CG8167, CG4019, CG1944, CG5577, CG12142, CG8186, CG9390, CG3348, CG18374, CG18104, CG7290, CG2680, CG8773, CG9848, CG5725, CG10091, CG10521, CG9396, CG3767, CG9879, CG8327, CG12173, CG1980, CG4546, CG10611, CG8206, CG8721, CG8481, CG18596, CG1140, CG10342, CG4501, CG10120, CG6371, CG14777, CG3140, CG2275, CG7161, CG9122, CG8996, CG6567, CG4070, CG1135, CG8415, CG17221, CG8381, CG4716, CG12129, CG4839, CG4994, CG10165, CG8993, CG2175, CG5059, CG2092, CG4918, CG1891, CG3714, CG10639, CG9674, CG12021, CG5497, CG18609, CG11796, CG6092, CG9334, CG13925, CG9547, CG1909, CG11892, CG3209, CG12239, CG7506, CG1874, CG4789, CG9354, CG8443, CG3671, CG9584, CG1524, CG9074, CG6684, CG9169, CG10099, CG5827, CG7361, CG9579, CG4285
12	CG6649, CG3573, CG7157, CG17022, CG4550, CG18284, CG10680, CG4294, CG7915, CG5962, CG11853, CG10096, CG2930, CG11842, CG9519, CG8343, CG7560, CG7084, CG17837, CG3239, CG9668, CG11073, CG7905, CG6910, CG14942, CG12558, CG6069, CG3088, CG5641, CG7300, CG3159, CG6117, CG7397, CG9137, CG17446, CG5253, CG6866, CG5433, CG8790, CG4104, CG4405, CG5778, CG17907, CG5711, CG3234, CG1497, CG17739, CG7106, CG17108, CG14935, CG1213

Figure S1. Clusters of *Drosophila* gene expression profiles $X_c(\tau)$. The clusters are obtained using the method described in section 2b. For each cluster *c*, the average profile $X_c(\tau)$ is indicated in white.

(a) 10 clusters of the embryonic time series

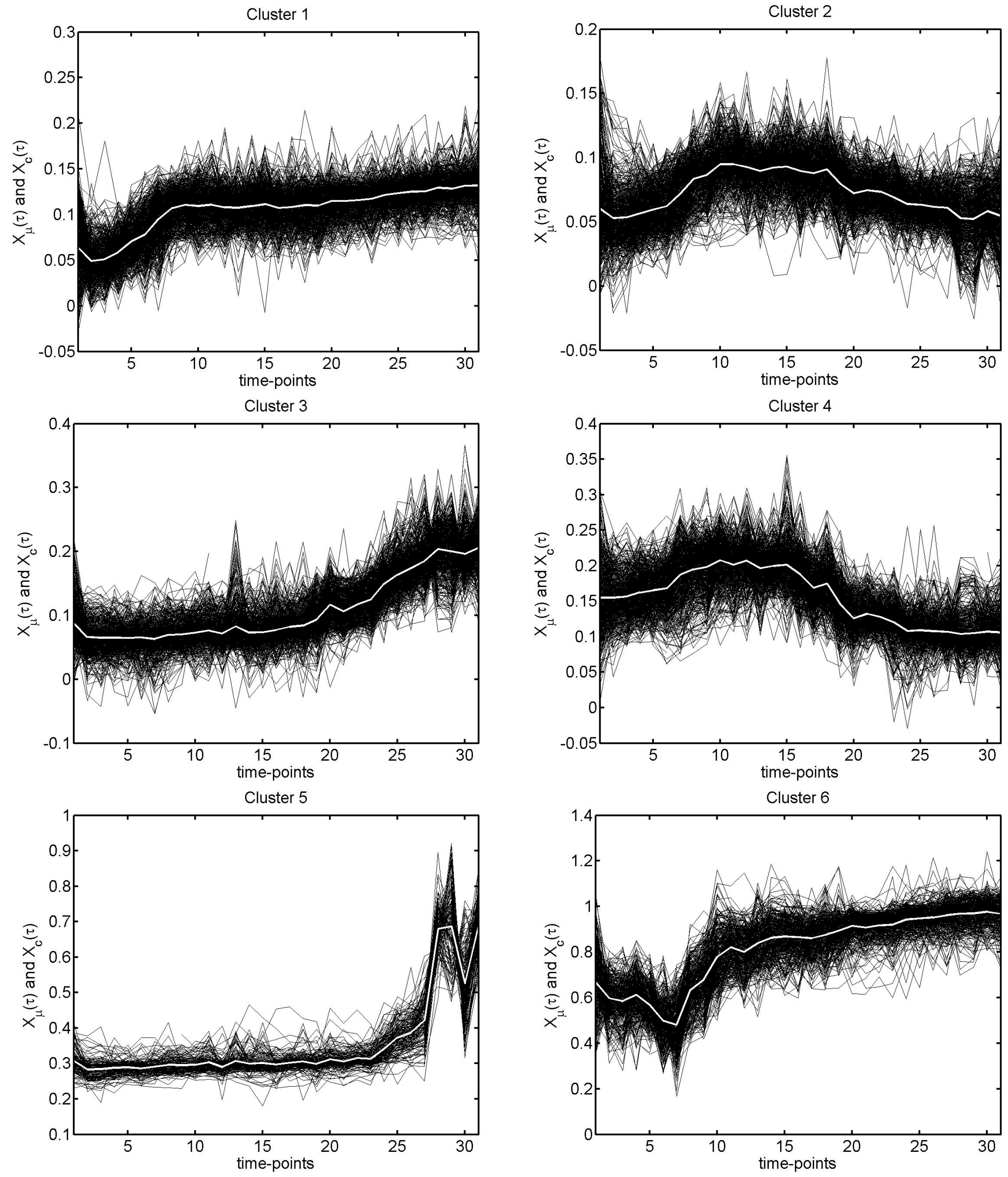

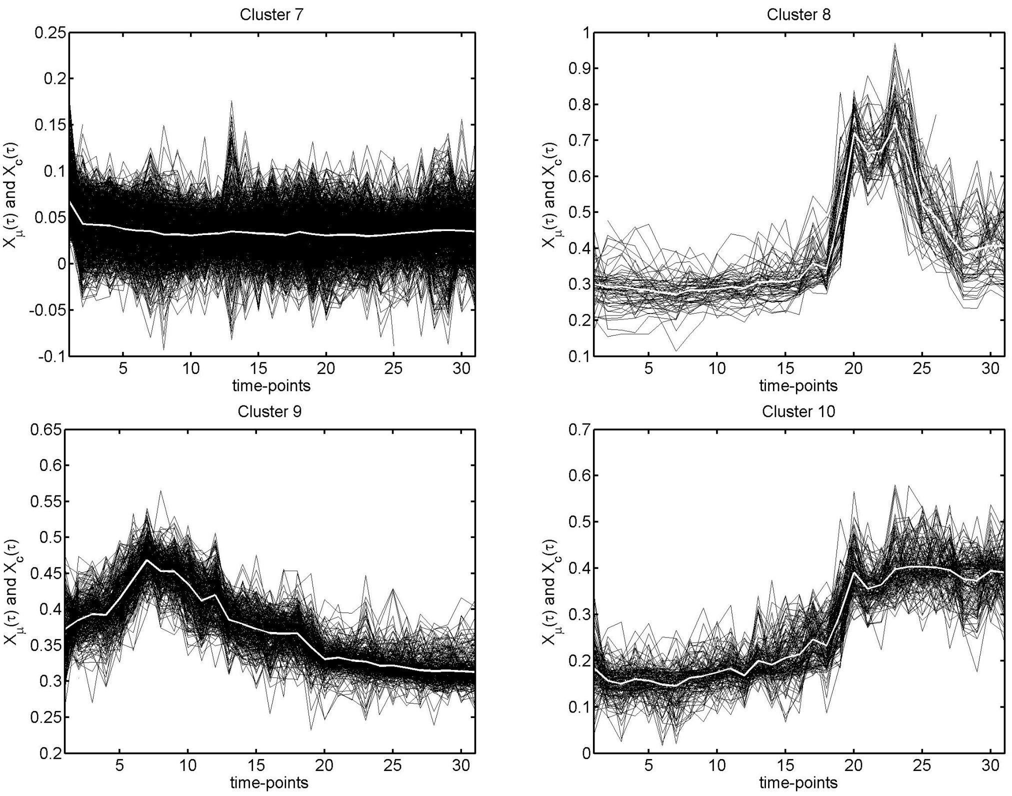

(b) 12 clusters of the full time series

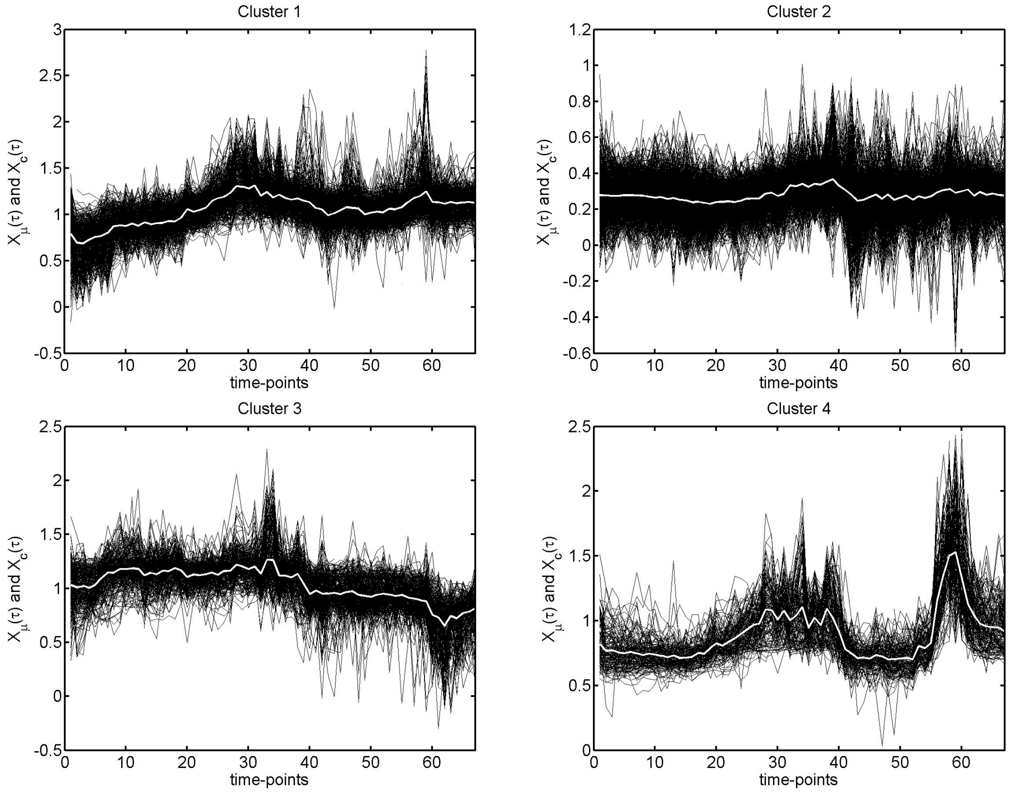

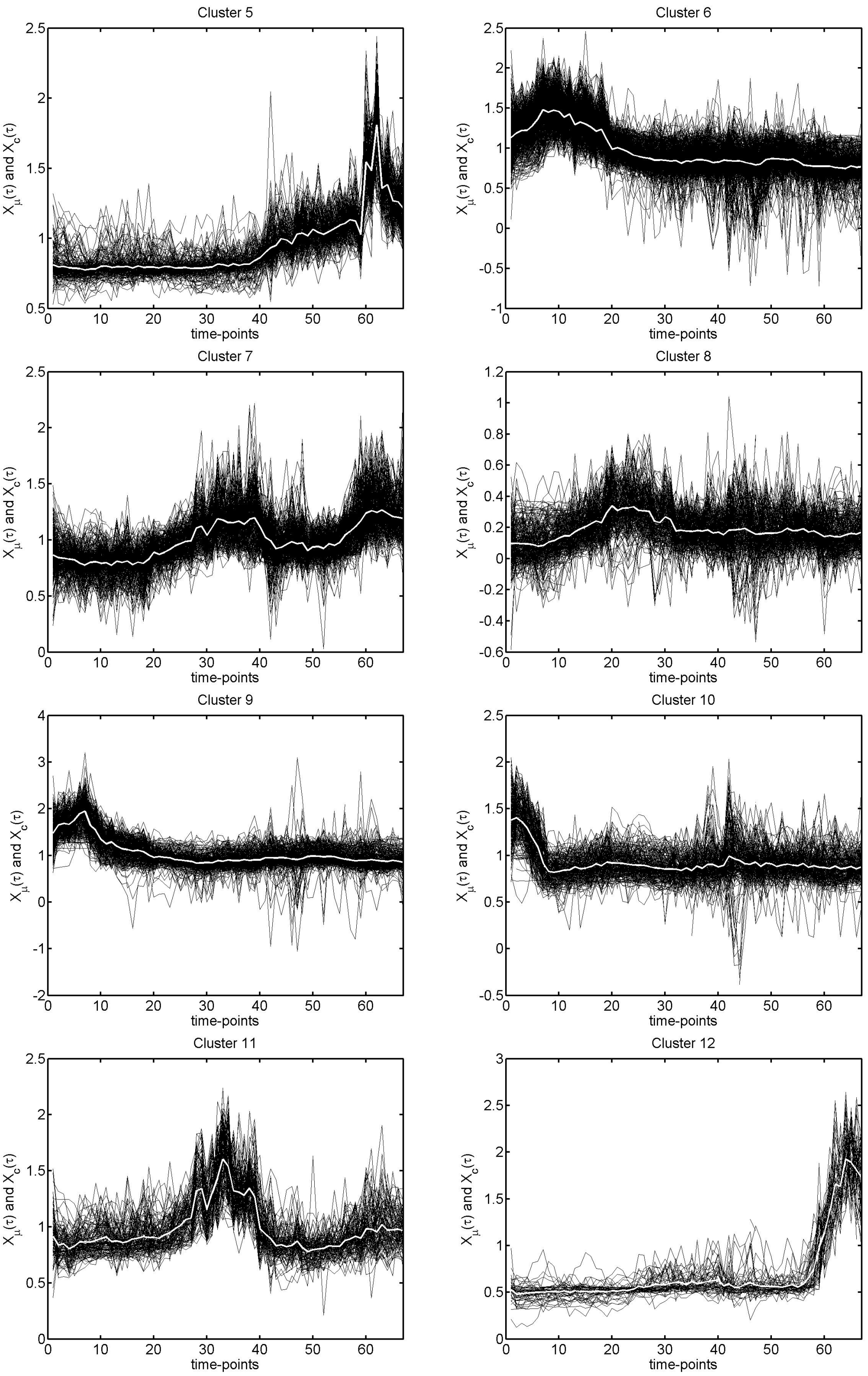
Cluster 5
Cluster 6
Cluster 7
Cluster 8
Cluster 9
Cluster 10
Cluster 11
Cluster 12
Xμ(τ) and Xc(τ)
time-points

Figure S2. Criteria for evaluating the quality of the estimated gene expression profiles upon parameter reduction. Standard deviation σ, standard deviation upon perturbation σ_{pert} and stability upon extrapolation in time χ, as a function of the number of parameters *p*, for the different model structures considered. Dashed-dotted line: reduction procedure Ψ_{σ} (see section 2.f); thin solid line: Ψ_{v}; dotted line: Ψ_{F}^{-}; dashed line: Ψ_{F}^{+}; thick solid line: Ψ_{P}. The unreduced solution is indicated with a black circle. For each reduction procedure, the most reduced solution that satisfies $\sigma_c \leq 0.5$ ∀c, where σ_c is defined in eq. (11), are indicated with a white circle. Note that some unreduced models do not satisfy this condition ($m_{NC}^{\exp}$ for the embryonic time series and $m_{CN}^{\exp}$ for the full time series) and are thus not shown.

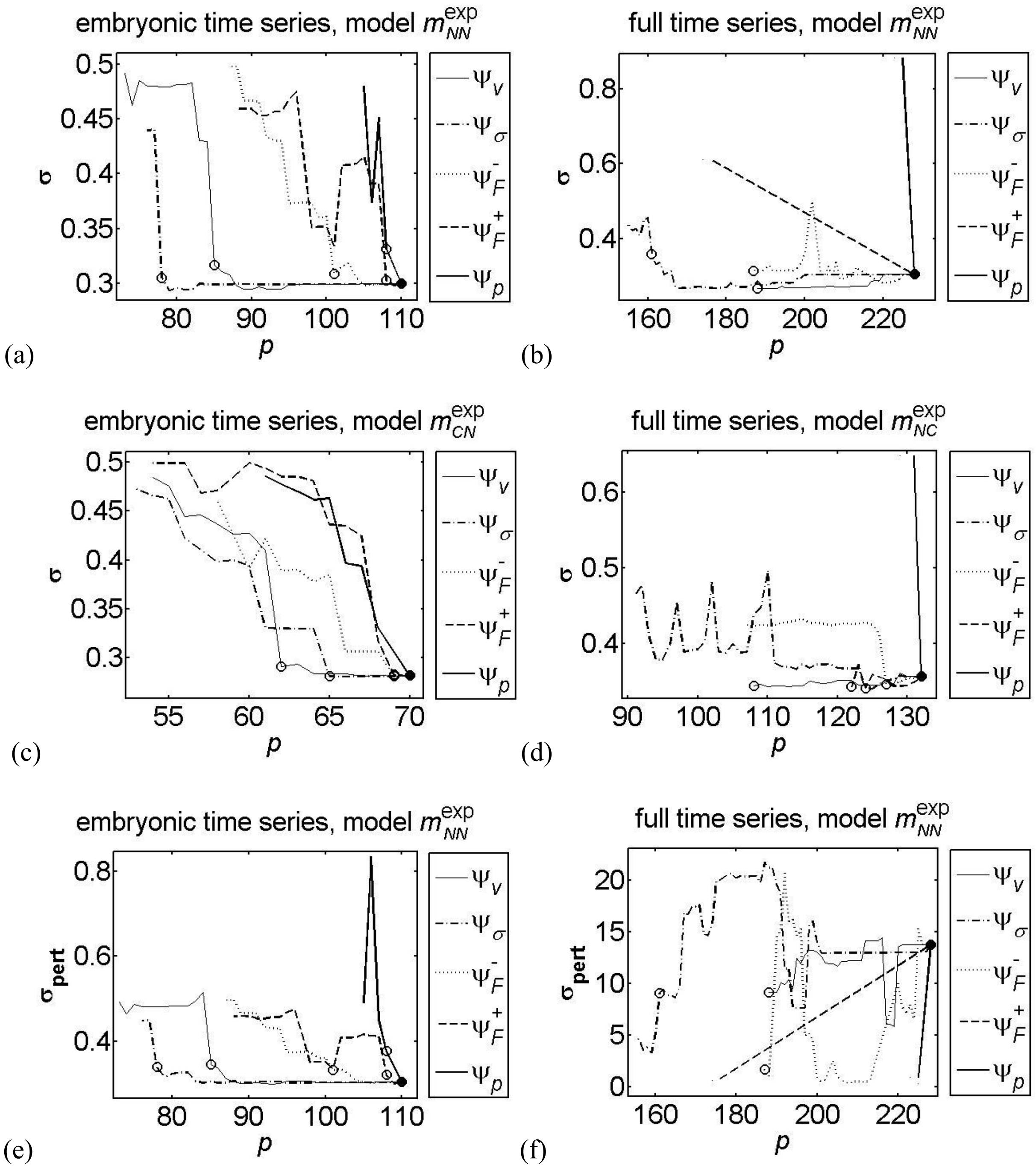

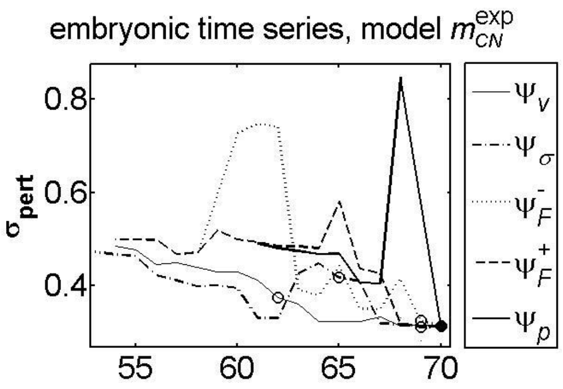
embryonic time series, model m_{CN}^{exp}
σ_{pert}
0.4
0.6
0.8
55
60
65
70
p
Ψ_v
Ψ_σ
Ψ_F^-
Ψ_F^+
Ψ_p

(g)

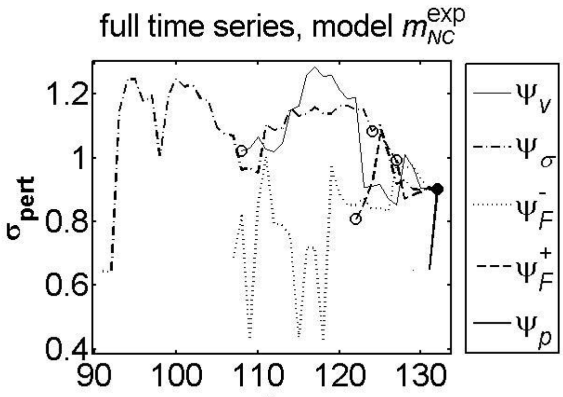
full time series, model m_{NC}^{exp}
σ_{pert}
0.4
0.6
0.8
1
1.2
90
100
110
120
130
p
Ψ_v
Ψ_σ
Ψ_F^-
Ψ_F^+
Ψ_p

(h)

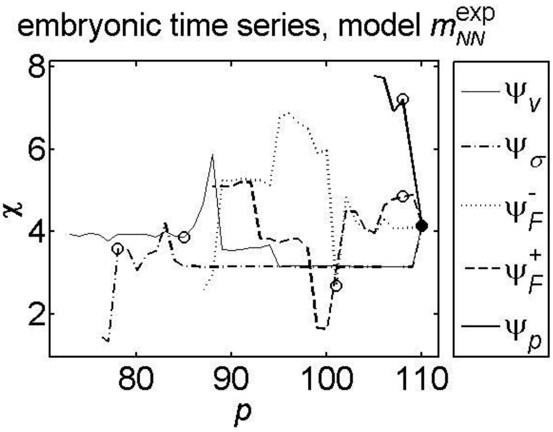
embryonic time series, model m_{NN}^{exp}
χ
2
4
6
8
80
90
100
110
p
Ψ_v
Ψ_σ
Ψ_F^-
Ψ_F^+
Ψ_p

(i)

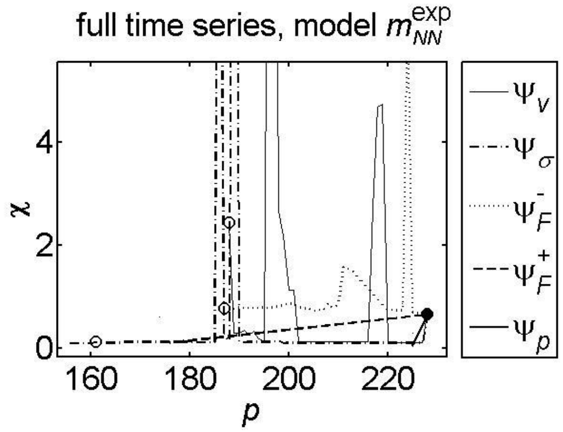
full time series, model m_{NN}^{exp}
χ
0
2
4
160
180
200
220
p
Ψ_v
Ψ_σ
Ψ_F^-
Ψ_F^+
Ψ_p

(j)

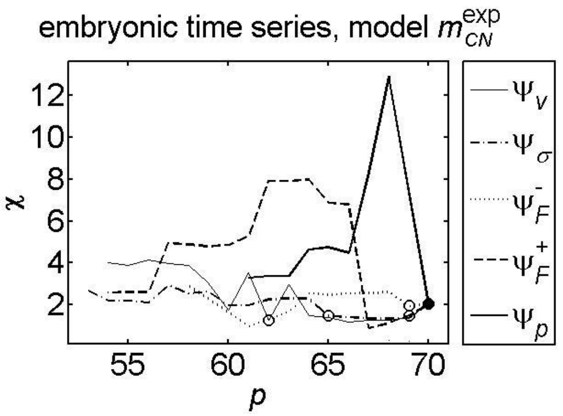
embryonic time series, model m_{CN}^{exp}
χ
2
4
6
8
10
12
55
60
65
70
p
Ψ_v
Ψ_σ
Ψ_F^-
Ψ_F^+
Ψ_p

(k)

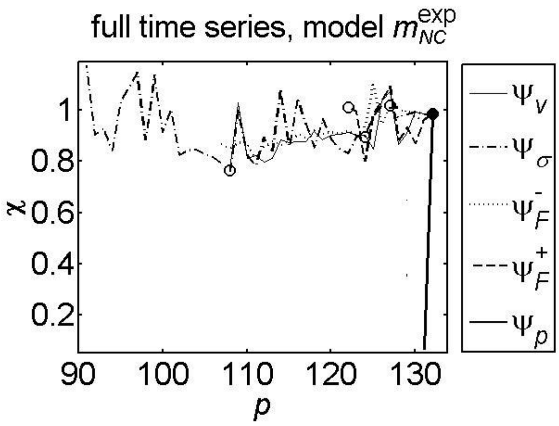
full time series, model m_{NC}^{exp}
χ
0.2
0.4
0.6
0.8
1
90
100
110
120
130
p
Ψ_v
Ψ_σ
Ψ_F^-
Ψ_F^+
Ψ_p

(l)

Figure S3. Experimental ($\bar{X}_c$) and estimated ($\hat{\bar{X}}_c$) gene expression profiles obtained with the two selected reduced solutions for the embryonic stage. The curves are given as a function of the real time. Dots: experimental data ; solid line: model structure $m_{NN}^{\exp}$ with the reduction scheme Ψ_σ ; dashed line: model structure $m_{CN}^{\exp}$ with the reduction scheme Ψ_v ; (a-j) clusters 1 to 10.

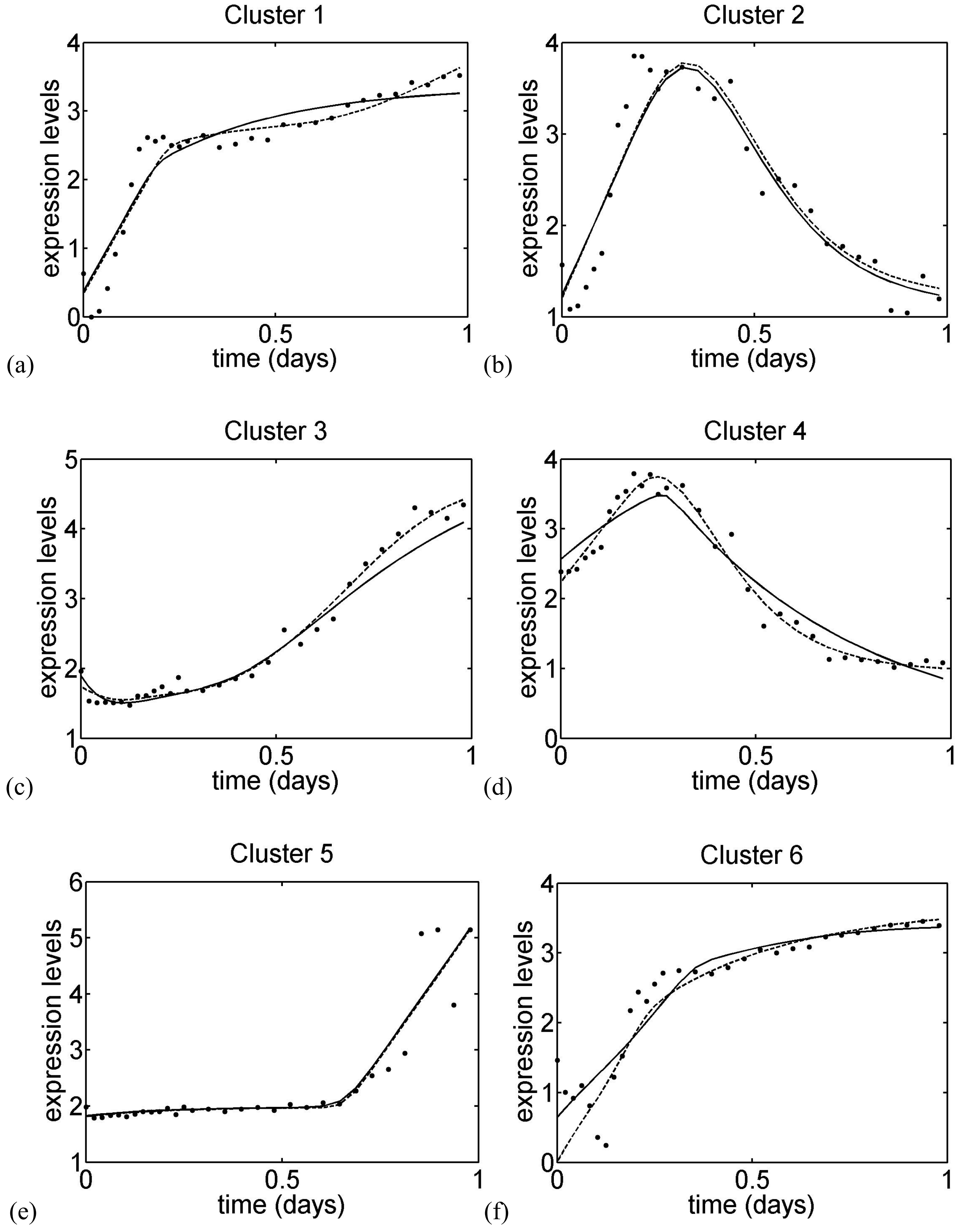

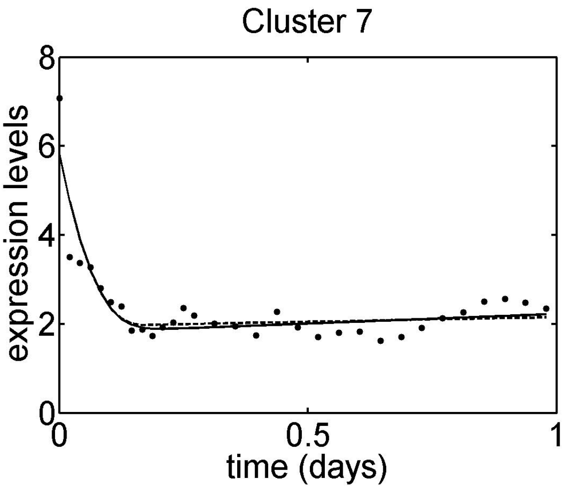
Cluster 7
expression levels
time (days)
0
2
4
6
8
0.5
1

(g)

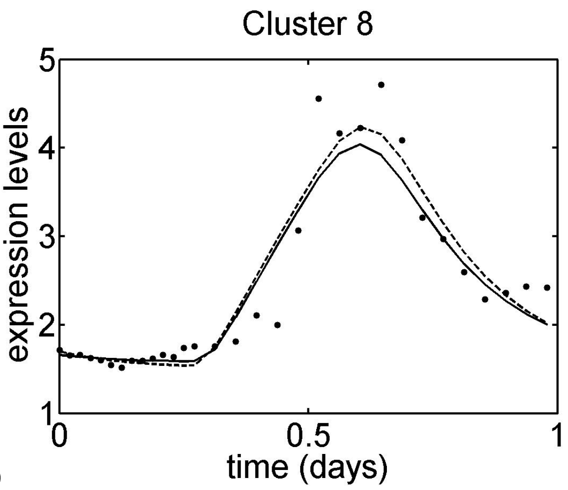
Cluster 8
expression levels
time (days)
1
2
3
4
5
0
0.5
1

(h)

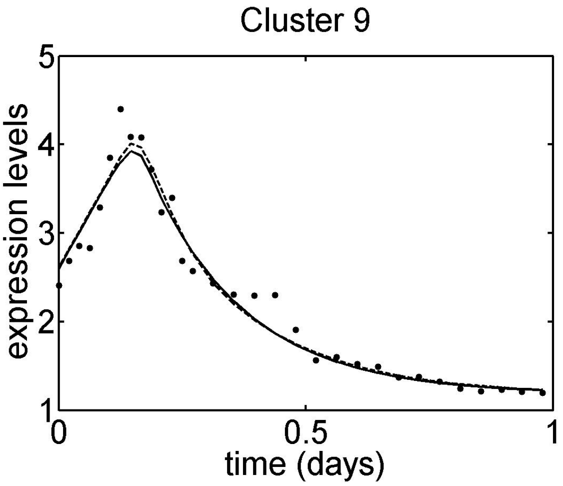
Cluster 9
expression levels
time (days)
1
2
3
4
5
0
0.5
1

(i)

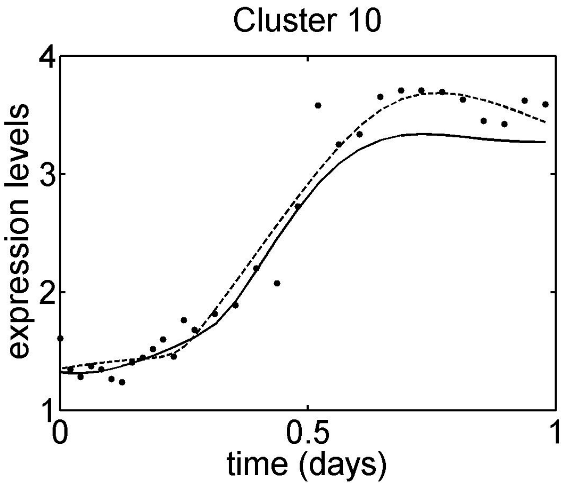
Cluster 10
expression levels
time (days)
1
2
3
4
0
0.5
1

(j)

Figure S4. Experimental ($\bar{X}_c$) and estimated ($\hat{\bar{X}}_c$) gene expression profiles obtained with the two selected reduced solutions for the full time series. The curves are given as a function of the time points. Dots: experimental data; solid line: model structure $m_{NN}^{\exp}$ with the reduction scheme Ψ_F^-; dashed line: model structure $m_{NN}^{\exp}$ with the reduction scheme Ψ_v; (a-l) clusters 1 to 12.

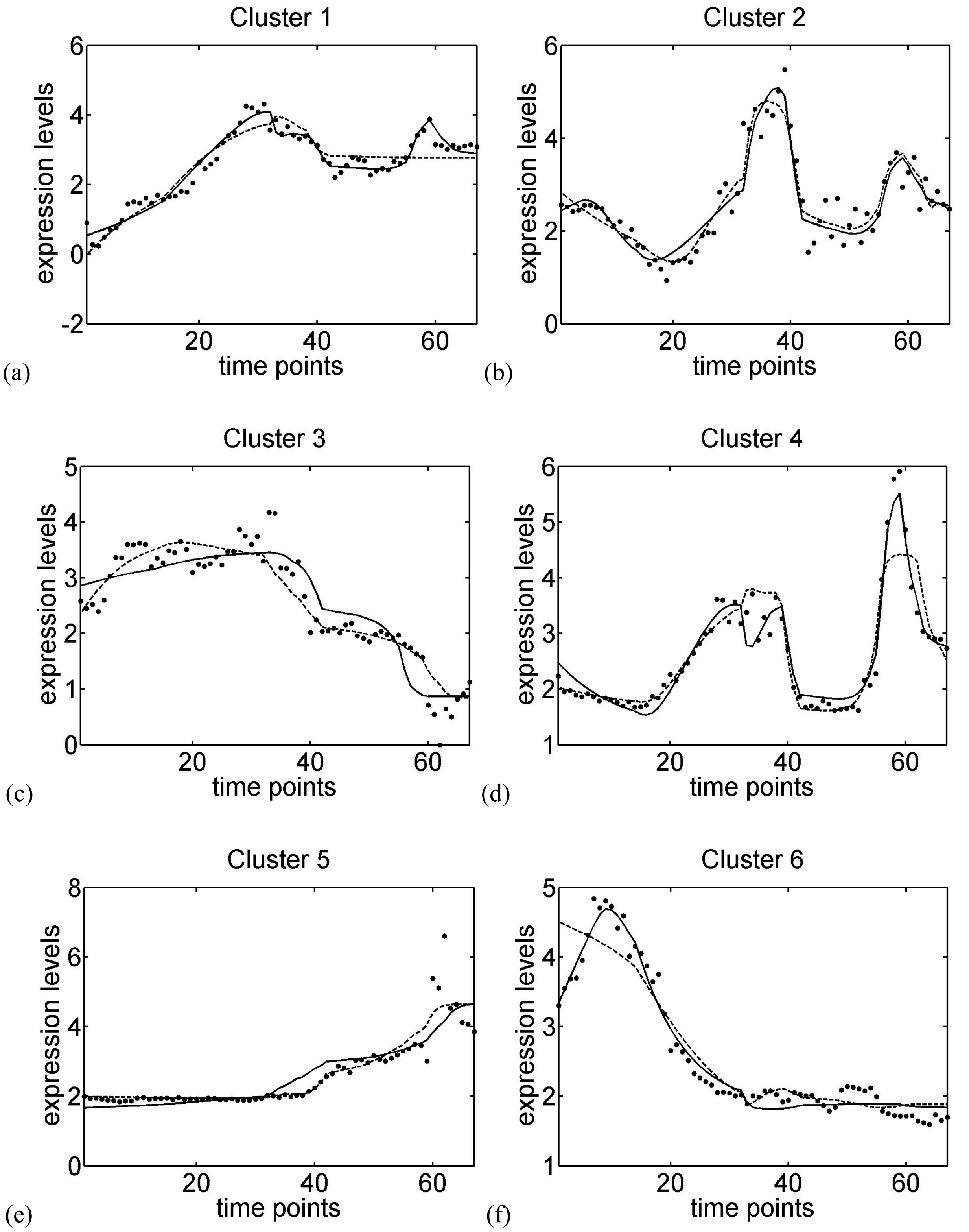

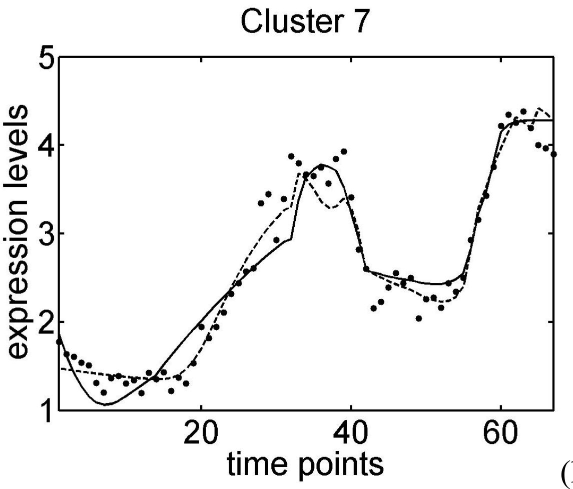

Cluster 7
expression levels
time points
1
2
3
4
5
20
40
60

(g)

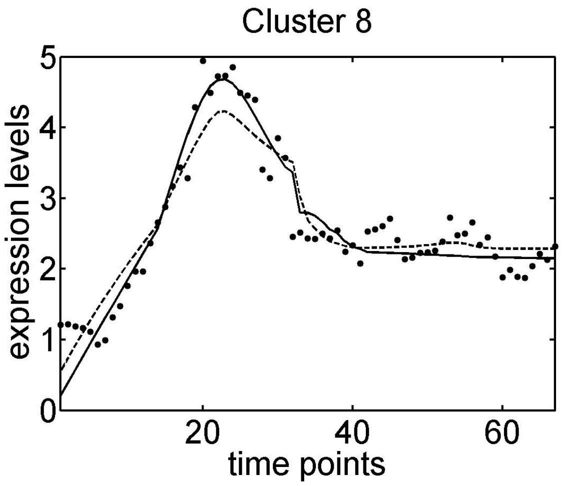

Cluster 8
expression levels
time points
0
1
2
3
4
5
20
40
60

(h)

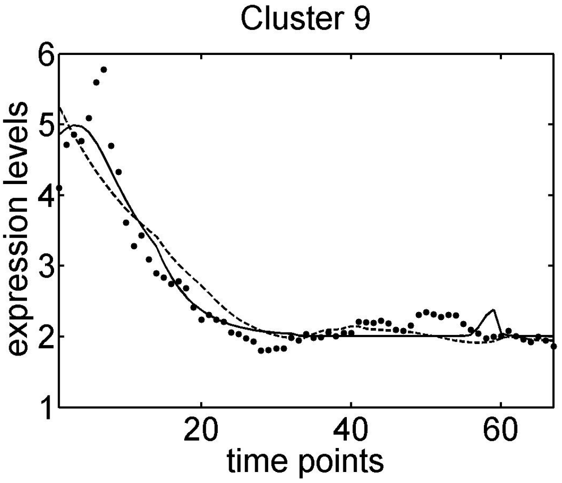

Cluster 9
expression levels
time points
1
2
3
4
5
6
20
40
60

(i)

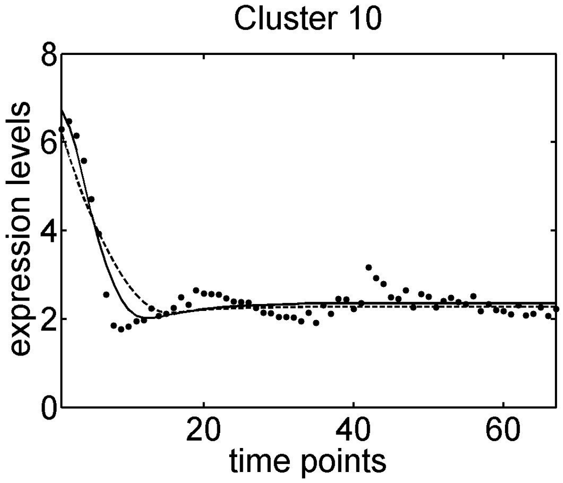

Cluster 10
expression levels
time points
0
2
4
6
8
20
40
60

(j)

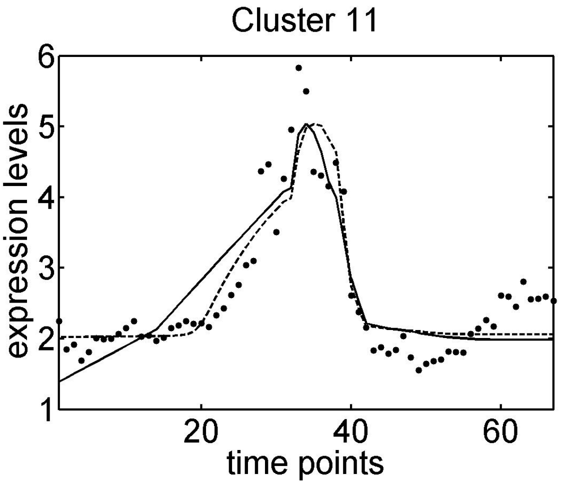

Cluster 11
expression levels
time points
1
2
3
4
5
6
20
40
60

(k)

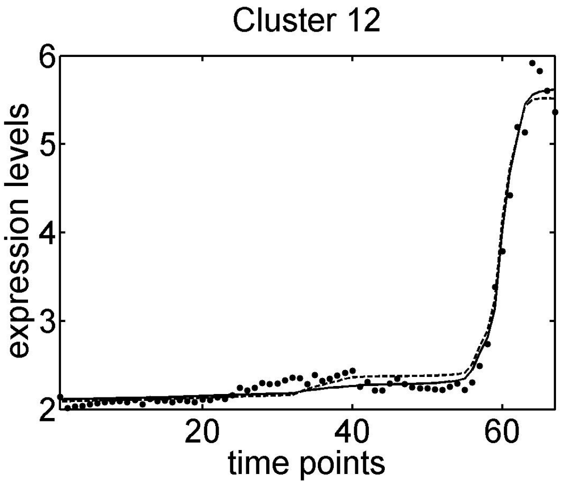

Cluster 12
expression levels
time points
2
3
4
5
6
20
40
60

(l)

Figure S5. Gene regulatory network corresponding to selected reduced solutions for the full time series. The best reduced solutions obtained with the model structures $m_{NN}^{\exp}$ and $m_{NC}^{\exp}$ for the full time series, as defined by a good reproduction of the data, a low number of parameters, a good robustness with respect to parameter variations and a good stability in time (corresponding to the gray lines in Table 1b). The symbols “1” and ”-1” represent activation and inhibition, respectively. (a) Network obtained with the model structure $m_{NC}^{\exp}$ and the reduction scheme Ψ_F^-; (b) Network obtained with the model structure $m_{NC}^{\exp}$ and the reduction scheme Ψ_v.

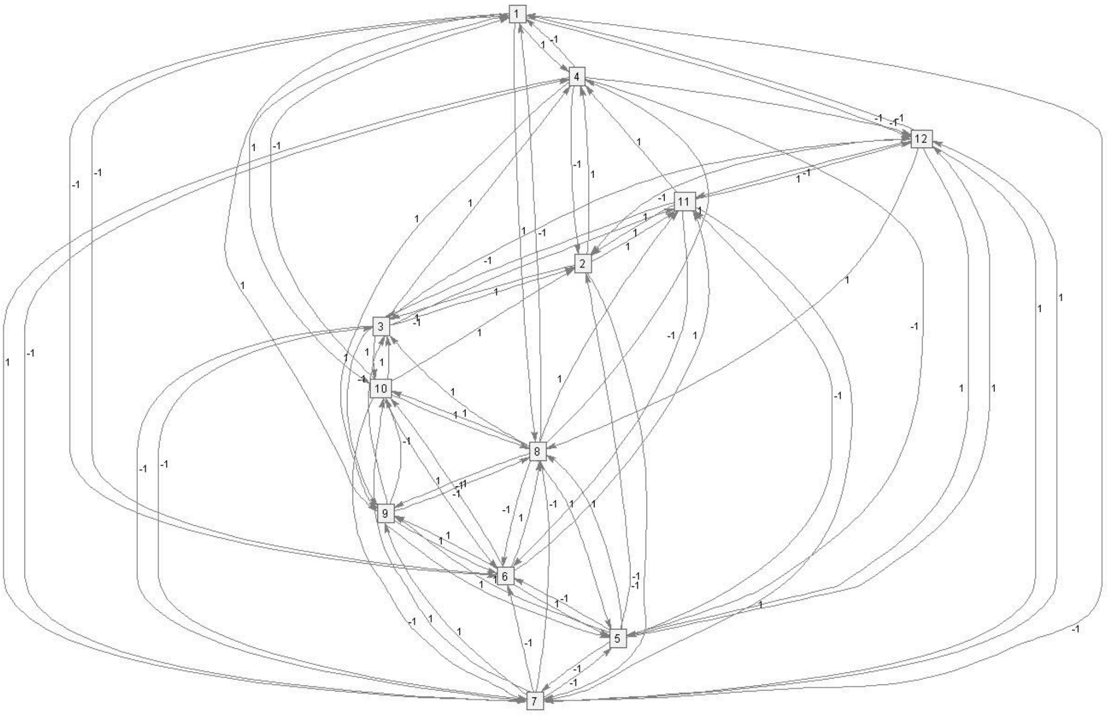

(a)

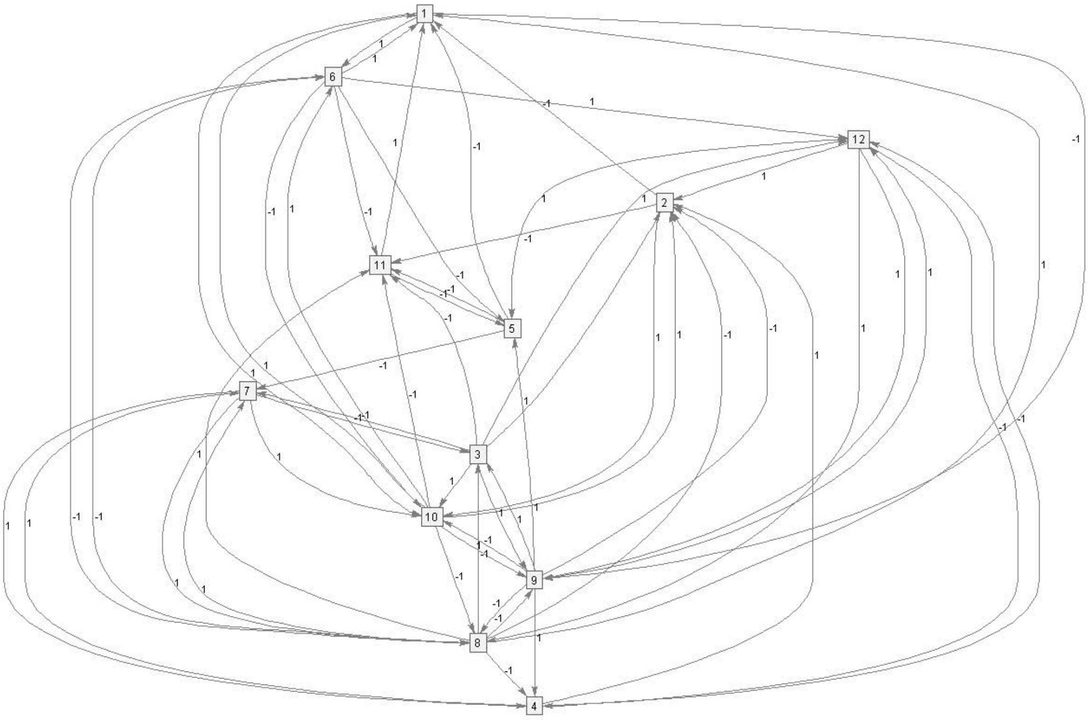

(b)
